# Supplementary material for: Controllable deuteration of halogenated compounds by photocatalytic D2O splitting
Source: Nat Commun. 2018 Jan 8;9:80. doi: 10.1038/s41467-017-02551-8 (PMC5758826; doi:10.1038/s41467-017-02551-8)
Supplement: Supplementary file 1 — Supplementary Information [file 41467_2017_2551_MOESM1_ESM.docx]

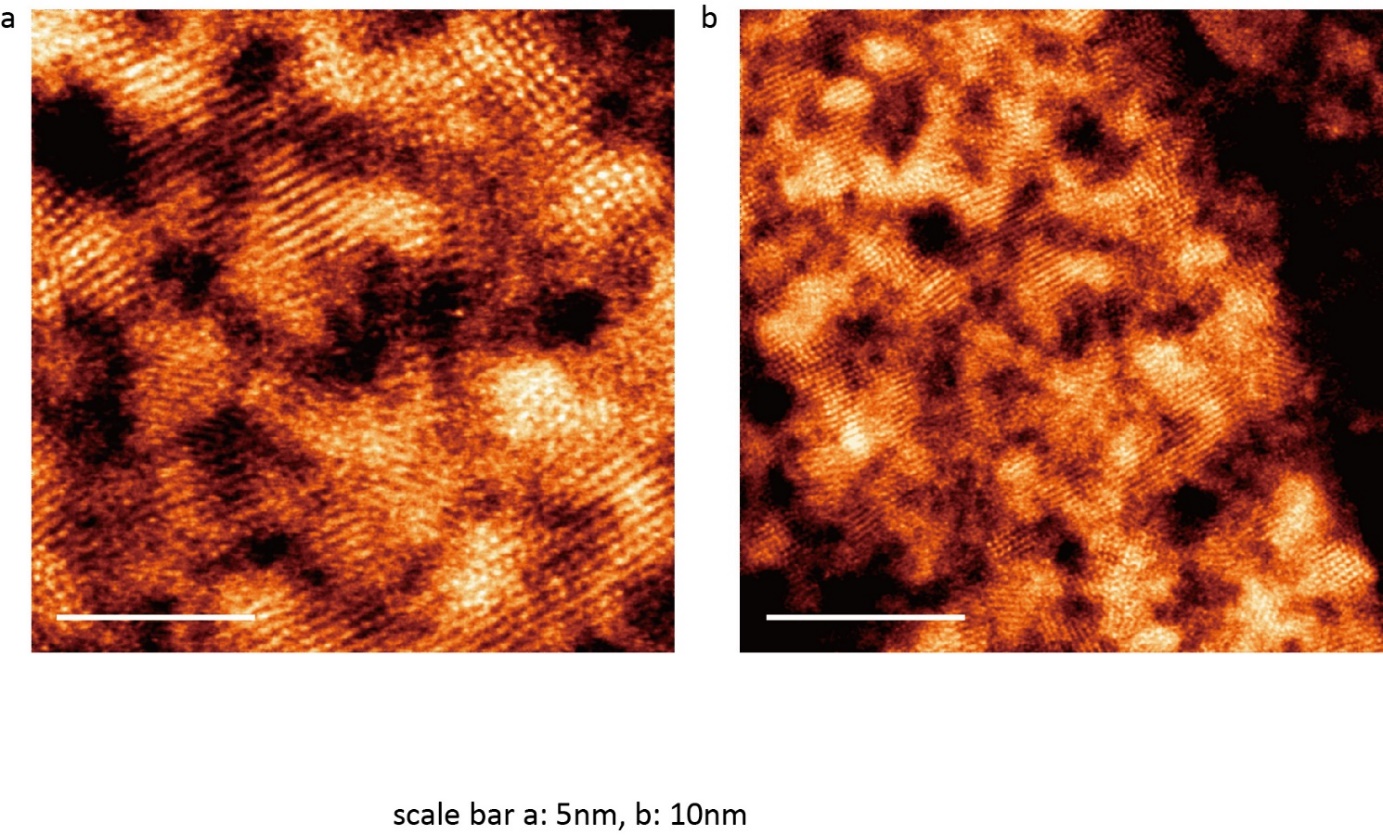


**Supplementary Figure 1.** High-resolution STEM-ADF images of porous CdSe nanosheets, revealing the crystalline nanodomains, amorphous domains as well as nanopores on CdSe. Scale bar: a. 5 nm; b. 10 nm. The porosity of CdSe nanosheets is roughly 23 area%.


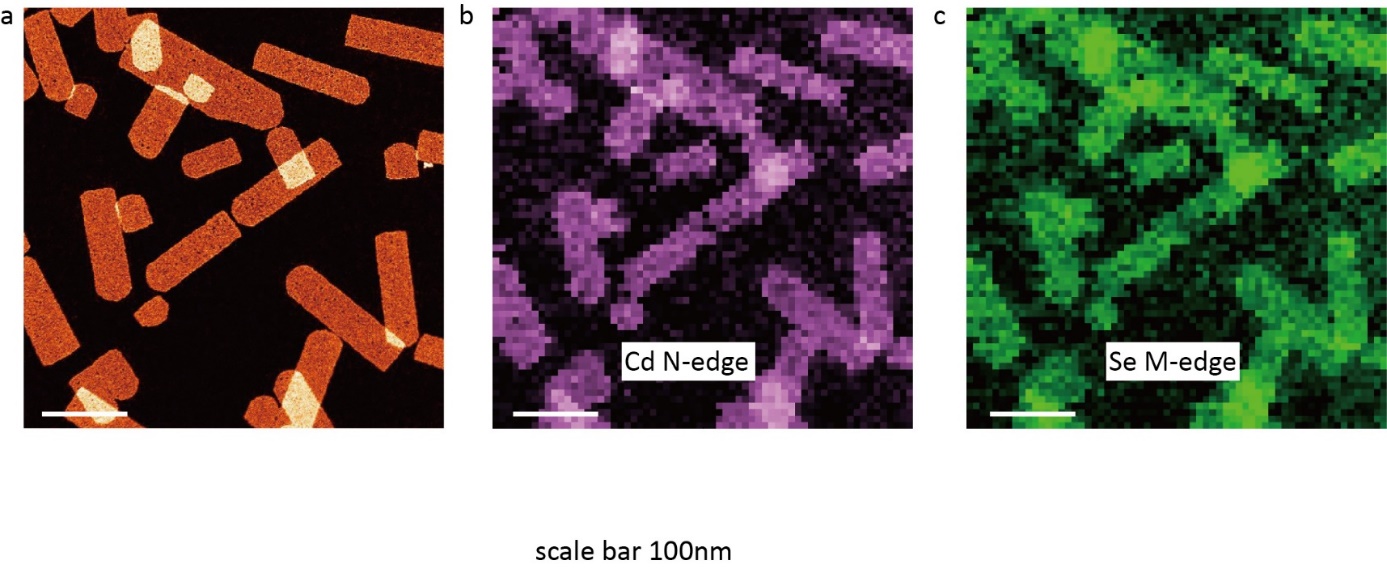


**Supplementary Figure 2.** STEM-ADF image and corresponding EELS mapping of porous CdSe nanosheets at a larger scale. Scale bar: a-c. 100 nm.


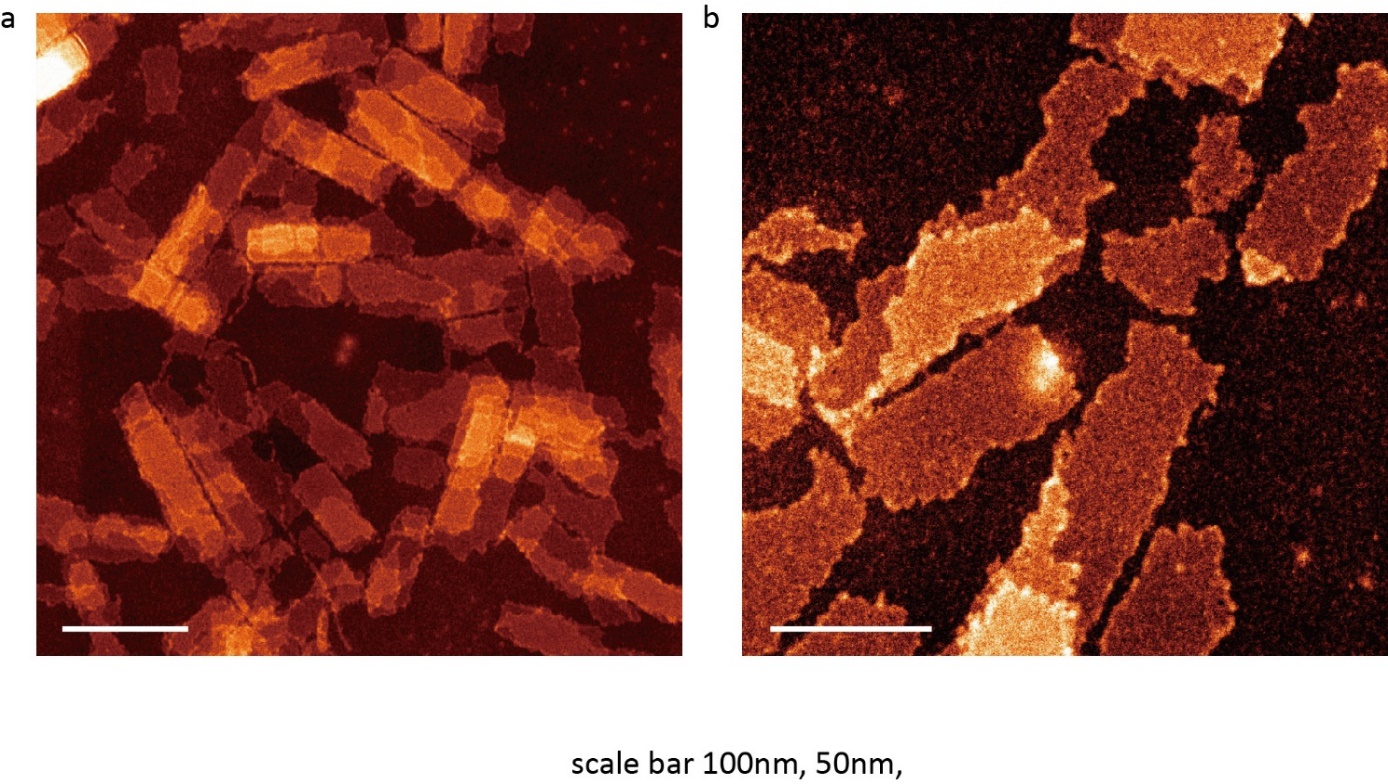


**Supplementary Figure 3.** STEM-ADF images of non-porous CdSe nanosheets. Scale bar: a. 100; b. 50 nm.


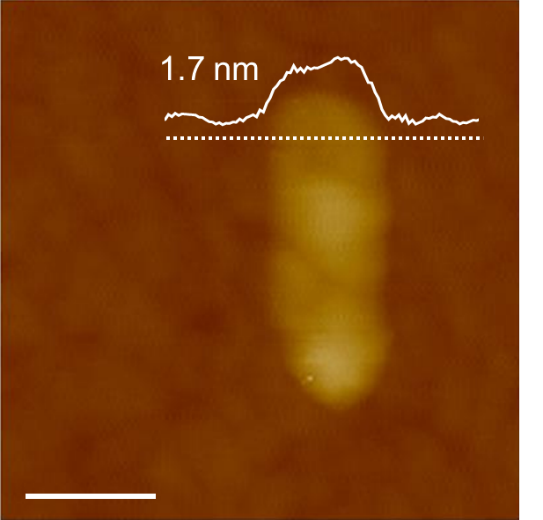


**Supplementary Figure 4.** Representative AFM image of porous CdSe nanosheets, showing its ultrathin layer structure with a thickness of 1.7 nm (~ seven layers). Scale bar: 50 nm. CdSe solution (~ 0.1 mg mL^-1^ in CHCl_3_) was spin-coated onto 300 nm SiO_2_/Si wafer prior to measurement. Scale bar: 50 nm.


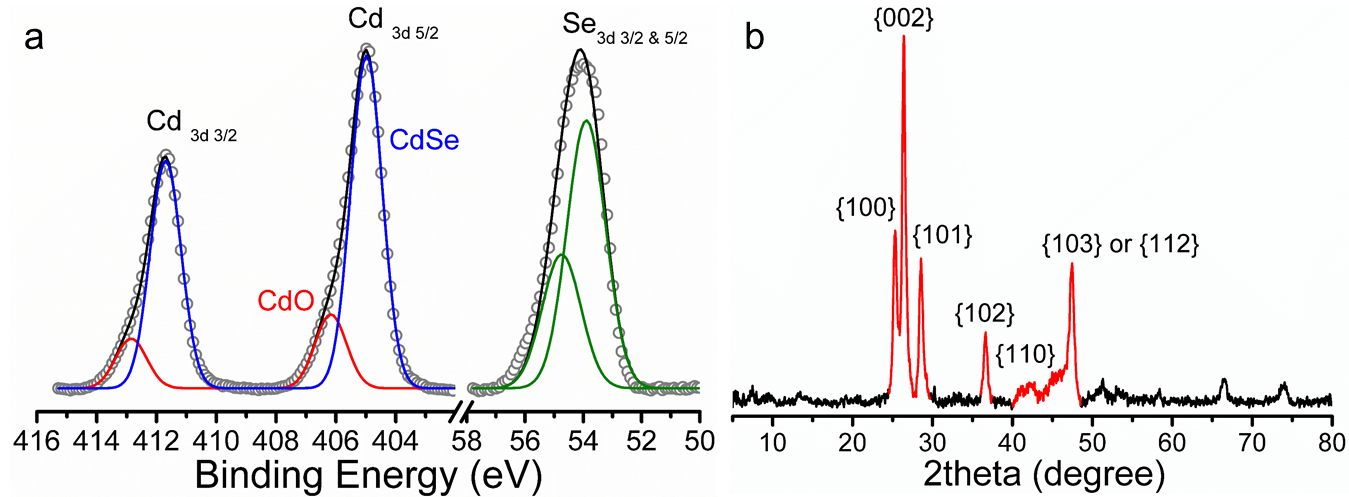


**Supplementary Figure 5.** (a) XPS Cd_3d_ / Se_3d_ spectra and (b) powder XRD pattern of porous CdSe nanosheets.





**Supplementary Figure 6.** Electrochemical bandgap measurements of 5 mM porous CdSe with 0.05 M tetrabutylammonium hexafluorophosphate (TBAPF_6_) and 0.4 mM ferrocene (Fc) in CH_3_CN. All potentials were calibrated to SHE using the Fc/Fc^+^ couple (0.470 V *vs.* SHE) as an internal standard^1^. HOMO level of porous CdSe was calculated from the onset oxidation potential (1.341 V *vs.* SHE)^2^.





**Supplementary Figure 7.** Large-scale synthesis of benzene (**2w’**) by the photocatalytic hydrodehalogenation.


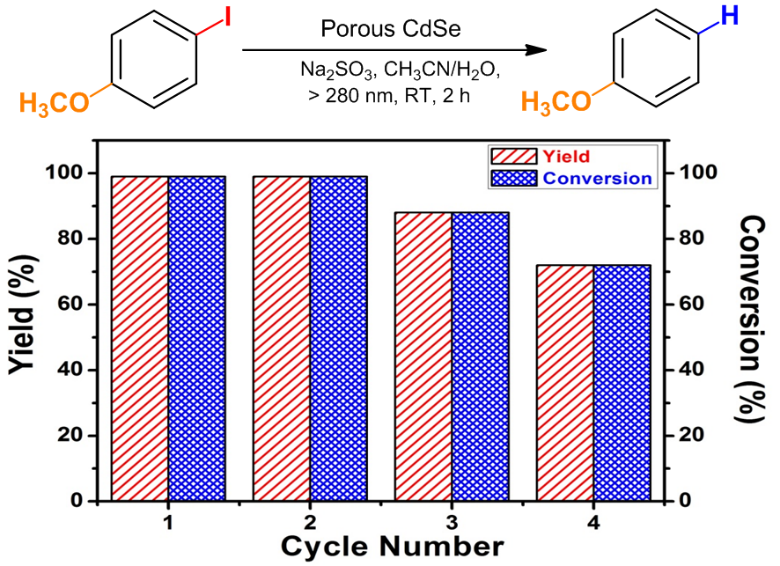


**Supplementary Figure 8.** Recycling efficiency of the porous CdSe nanosheets for photocatalytic hydrodehalogenation of halides. Each run was conducted with 0.1 mmol of *p*-iodoanisole (**1c**) under the optimized condition.





**Supplementary Figure 9.** Determination of hydrogen source from the hydrogenation/ deuteration of an iodinated substrate. The change in molecular weight from 160.0 to 161.1 in the presence of D_2_O suggests the hydrogen source is from water molecules. Control studies using deuterated acetonitrile show that the molecular weight does not change. The deuteration ratio is determined by NMR measurements of the isolated products.


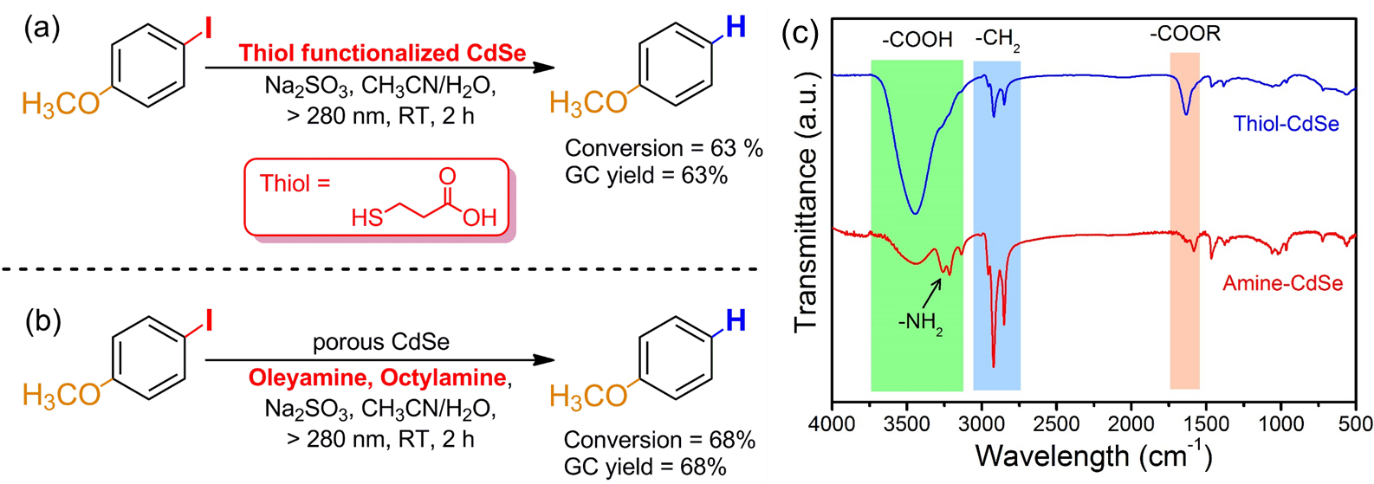


**Supplementary Figure 10.** Photocatalytic hydrodehalogenation of *p-*iodoanisol (**1c**) using (a) thiol functionalized, porous CdSe nanosheets (abbreviated as Thiol-CdSe) and (b) porous CdSe nanosheets in the presence of 10 mmol oleylamine and octylamine as sacrificial agents; (c) FT-IR spectra of Thiol-CdSe and Amine-CdSe (after recycled from solution). FT-IR spectra confirm the surface-passivation of active sites by thiol and amine molecules, leading to reduced conversion and GC yield for hydrodehalogenation.


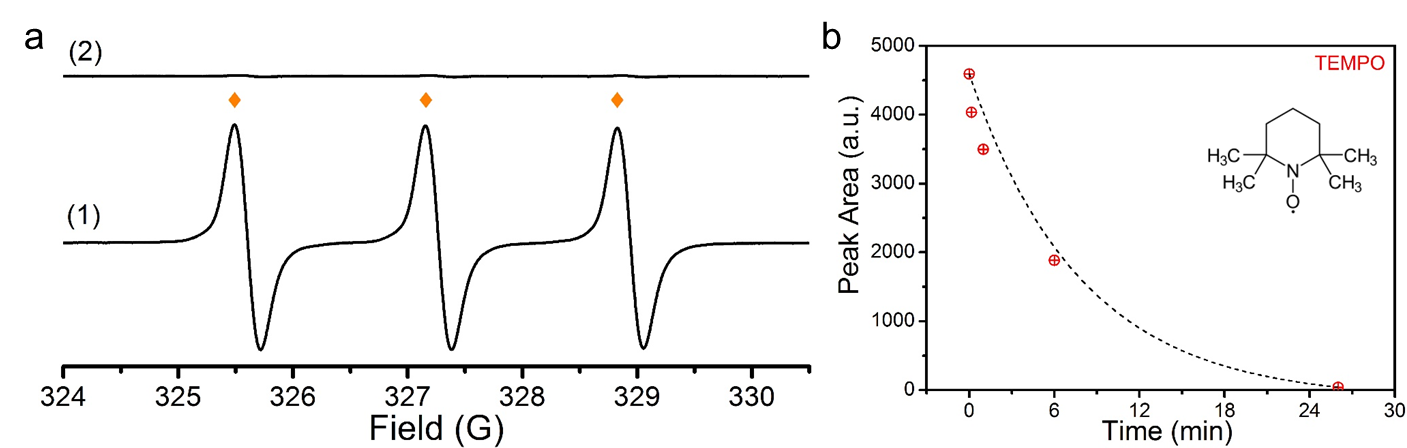


**Supplementary Figure 11.** (a) Quenching of TEMPO radical by photocatalytic hydrodehalogenation with 1 mM CdSe, 4 mM TEMPO, 2 mM *p*-iodoanisole (**1c**), 0.13 M Na_2_SO_3_ in (1:1 v/v) CH_3_CN/H_2_O: (1) in the dark, (2) after 26 mins irradiation of UV light (> 280 nm). (b) Time dependence of TEMPO quenching. ♦ is originated from TEMPO radical. Inset: chemical structure of TEMPO radical. The quenching of TEMPO radical suggests that radicals are produced in the photocatalytic reactions, and these react with TEMPO and deplete its concentration. We cannot rule out the possibility that TEMPO-R could also arise from the reduction of TEMPO• to TEMPO^-^ and subsequent protonation.


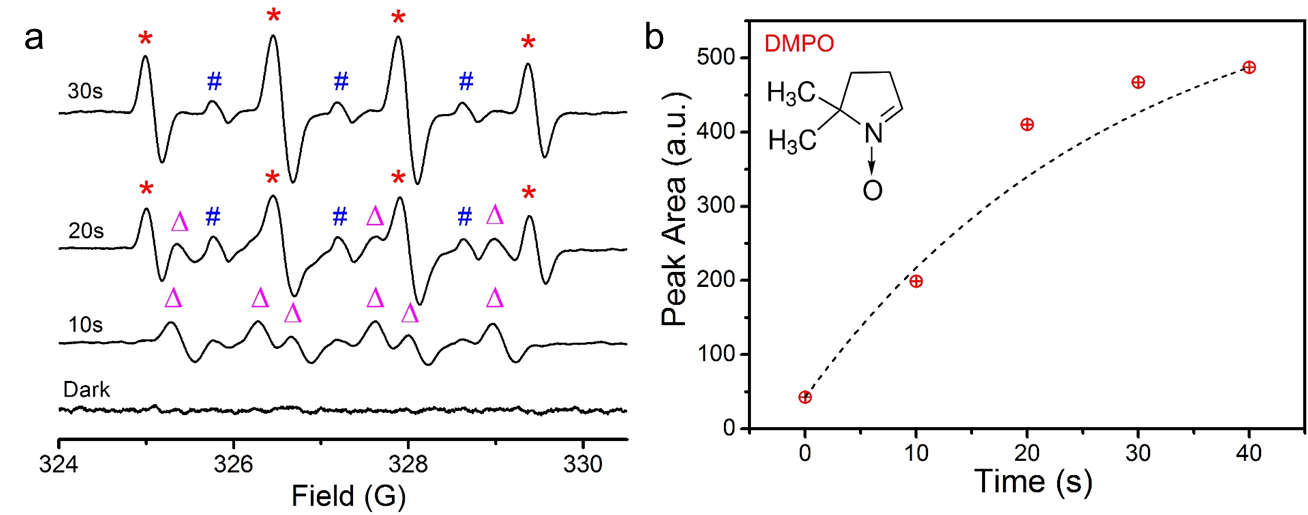


**Supplementary Figure 12.** (a) Trapping of DMPO-X (X = OOH, OH and C) adducts by photocatalytic hydrodehalogenation with 1 mM CdSe, 4 mM DMPO, 2 mM *p*-iodoanisole (**1c**), 0.13 M Na_2_SO_3_ in (1:1 v/v) CH_3_CN/H_2_O: (1) in dark, (2) after 10 s, (3) after 20 s and (4) after 30 s irradiation of UV light (> 280 nm). △, * and # represent the peaks from DMPO-OOH, DMPO-OH and DMPO-C radicals, respectively^3^. (b) Time dependence of DMPO trapping. Inset: chemical structure of DMPO molecule.


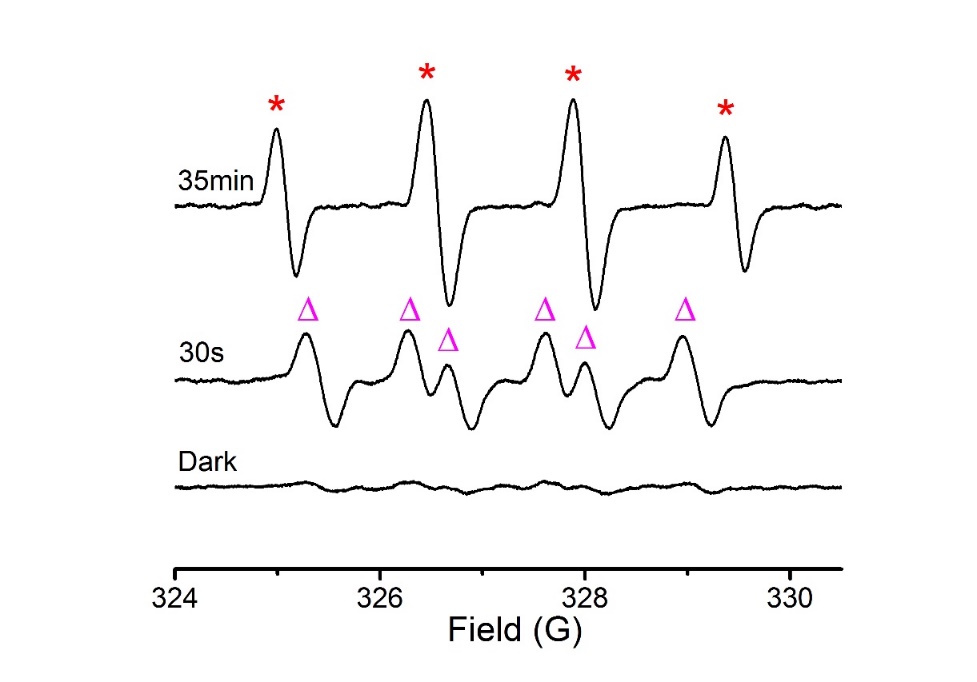


**Supplementary Figure 13.** (a) Trapping of DMPO-X (X = OOH & OH) adducts by photocatalytic hydrogen evolution with 1 mM CdSe, 4 mM DMPO, 0.13 M Na_2_SO_3_ in (1:1 v/v) CH_3_CN/H_2_O: (1) in the dark, (2) after 30 s and (3) after 35 mins irradiation of UV light (> 280 nm). △ and * represent the peaks from DMPO-OOH and DMPO-OH radicals. The signal of DMPO-OH gradually increases with time.


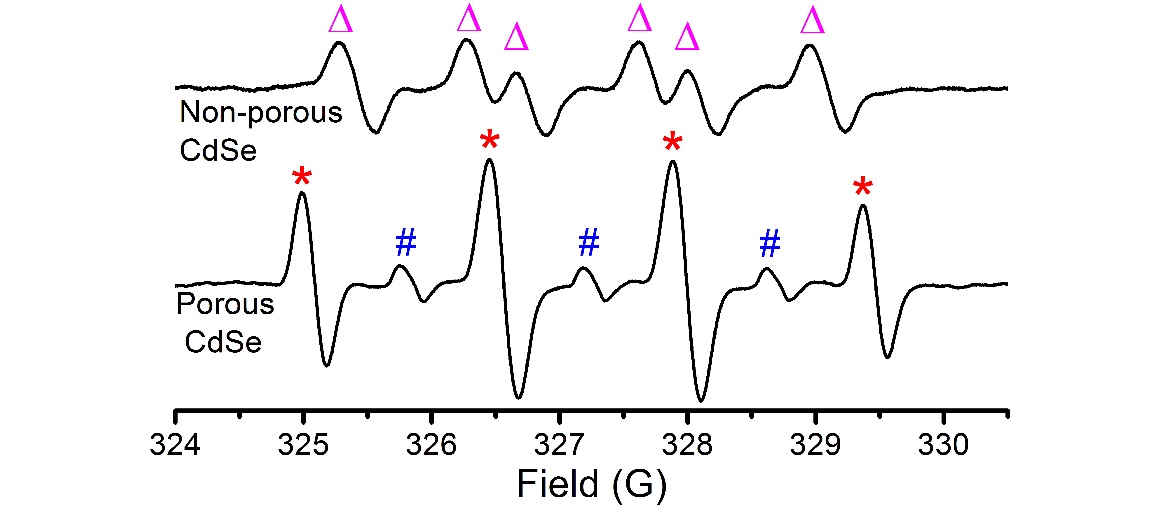


**Supplementary Figure 14.** Comparison in EPR signals of porous and non-porous CdSe nanosheets after 30 s irradiation of UV light (> 280 nm). Test condition is the same to Supplementary Figure 12. The vanishing of DMPO-OH and DMPO-C adducts in non-porous CdSe nanosheets indicates it has a much worse photocatalytic activity than porous nanosheets.





**Supplementary Figure 15.** PL spectra of porous CdSe nanosheets with/without the addition of iodobenzene.

**
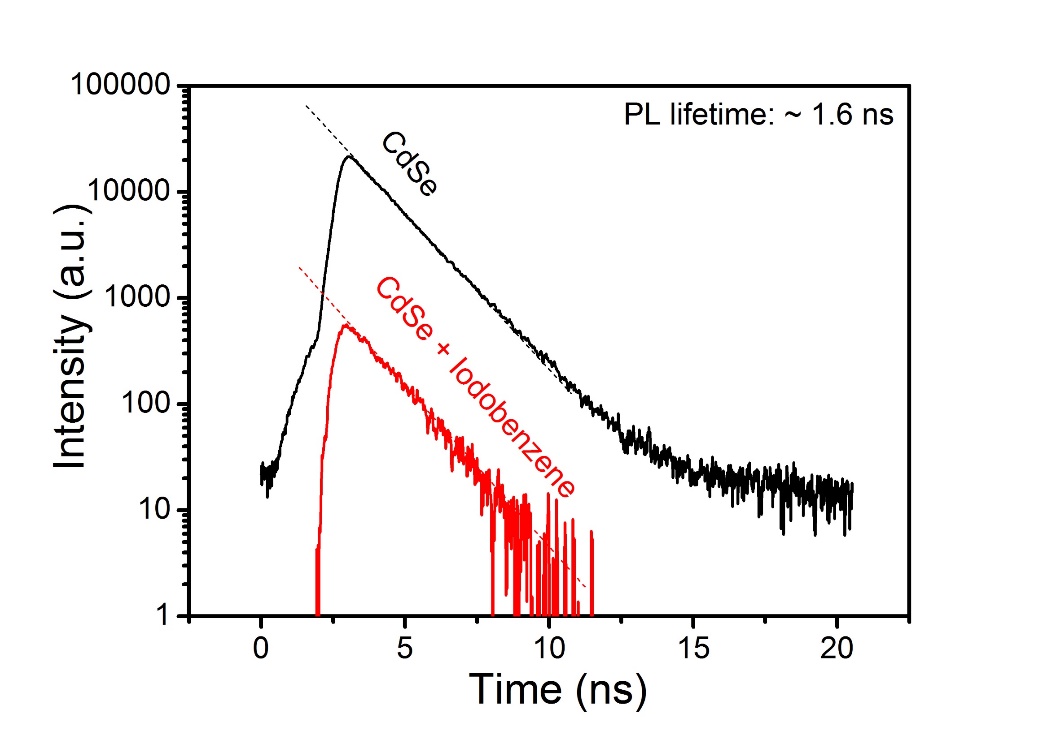
**

**Supplementary Figure 16.** PL lifetime semi-log plot of porous CdSe nanosheets with/ without the addition of iodobenzene. Excitation at 350 nm using ultrafast laser; emission at 437 nm. The PL lifetime (~ 1.6 ns) is estimated by the slope of semi-log plot for both samples. We can observe an obvious PL quenching after the addition of iodobenzene.





**Supplementary Figure 17.** Femtosecond transient absorption (TA) spectroscopy of porous CdSe nanosheets in CHCl_3_ without the addition of iodobenzene after the excition at 400 nm.





**Supplementary Figure 18.** Fitting curves of TA kinetics of porous CdSe in the absence and presence of iodobenzene. Pump: 400 nm, probe: 450 nm.


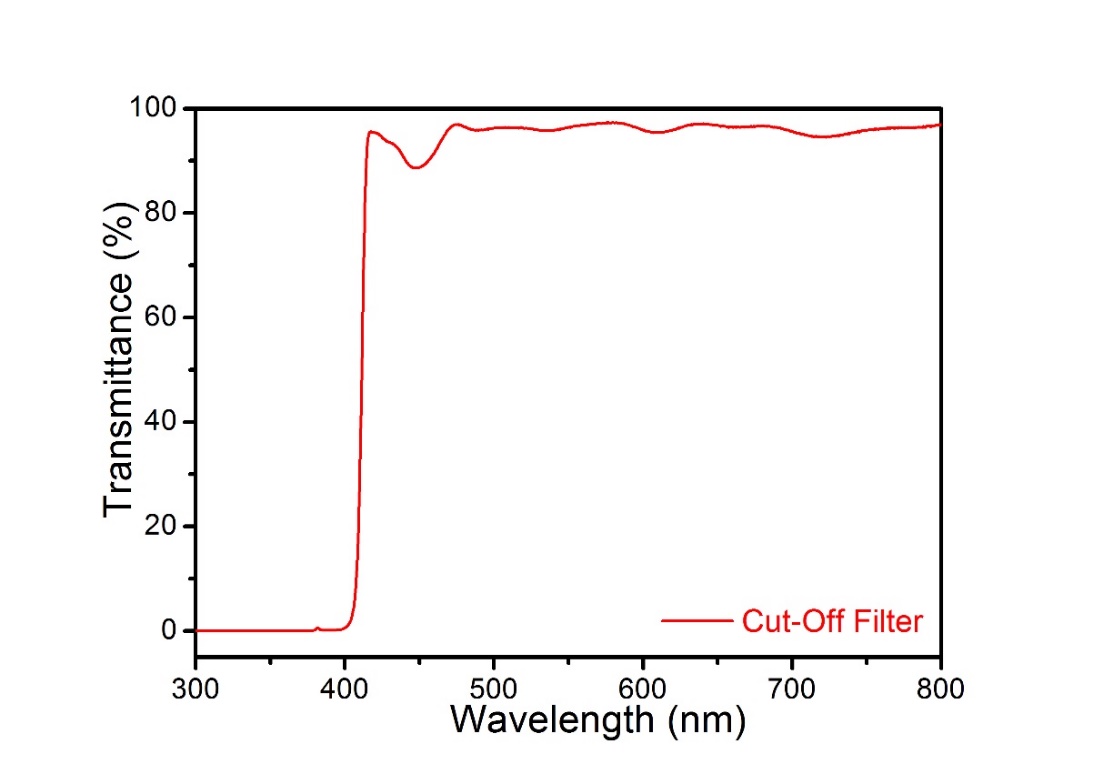


**Supplementary Figure 19.** UV-Vis spectrum of the cut-off filter (> 420 nm) in this study.


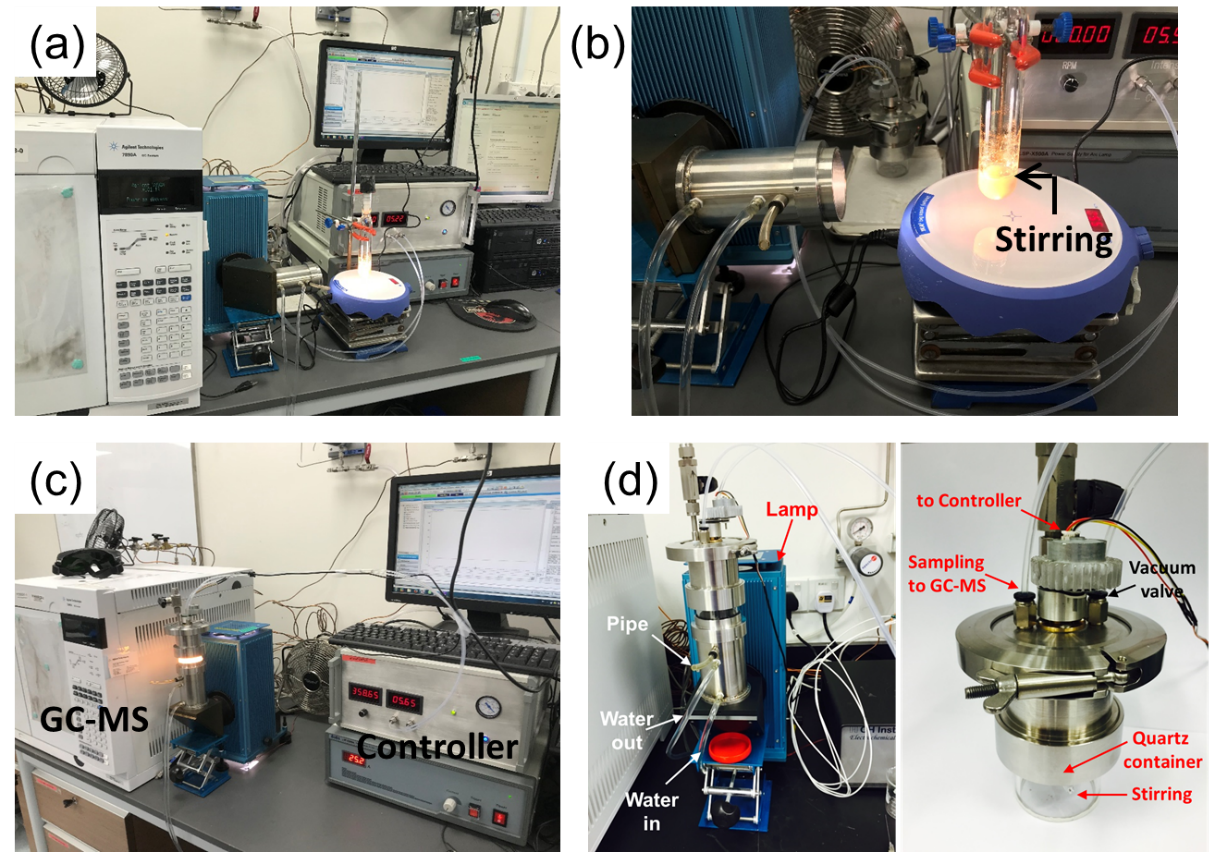


**Supplementary Figure 20.** Digital photos of the reaction setup. (a, b) Setup for hydrodehalogenation and deuteration. The lamp is ~ 10 cm away from the container; (c, d) Setup for photocatalytic water splitting with automatically sampling to GC-MS. The Xenon lamp (150 W, ~ 81 ± 2 mW cm^-2^ from light intensity meter) is water cooled to remove IR light. Selected cut-off filter can be installed between the lamp and reaction container.


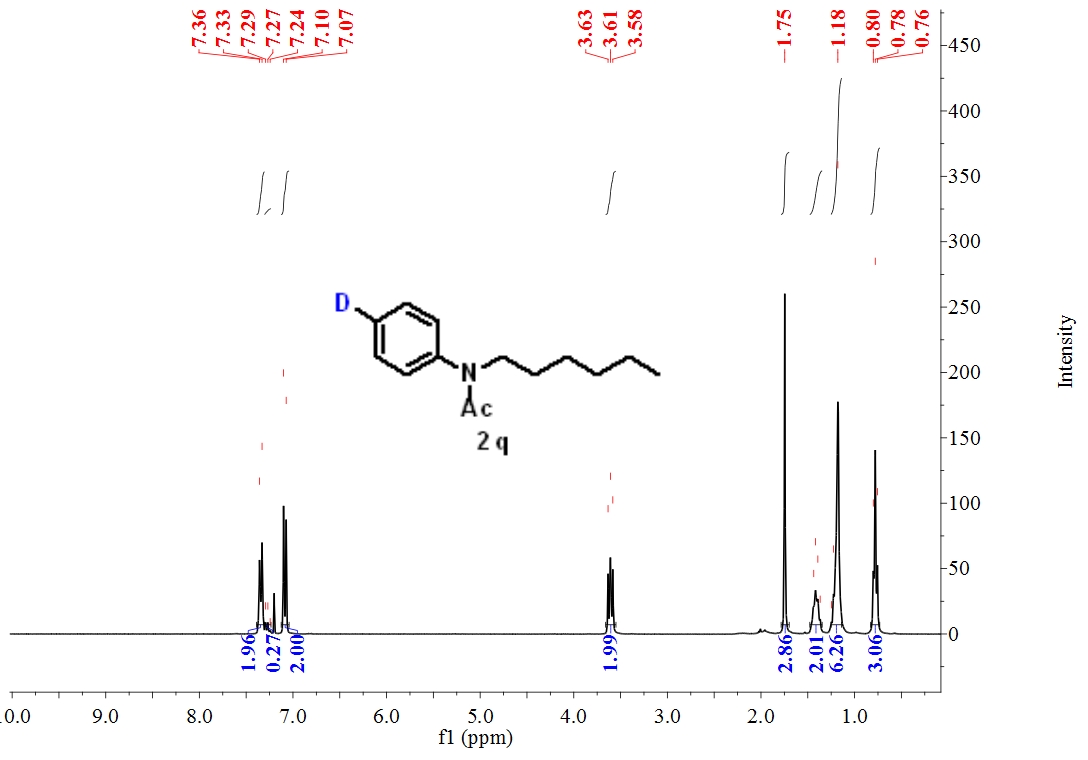


**Supplementary Figure 21.** ^1^H NMR of product **2q**. Deuterated ratio = 73%, **^1^H NMR** (300 MHz, Chloroform-*d*): *δ* [ppm] 7.34 (d, *J* = 8.0 Hz, 2H), 7.27 (t, *J* = 7.2 Hz, 0.27H), 7.09 (d, *J* = 8.3 Hz, 2H), 3.66 - 3.55 (m, 2H), 1.75 (s, 3H), 1.40 (q, *J* = 7.4, 6.5 Hz, 2H), 1.20 (d, *J* = 13.8 Hz, 6H), 0.83 - 0.73 (m, 3H); **HRMS** (APCI) 220.1689, theoretical value for C_14_H_20_DNO 220.1686.


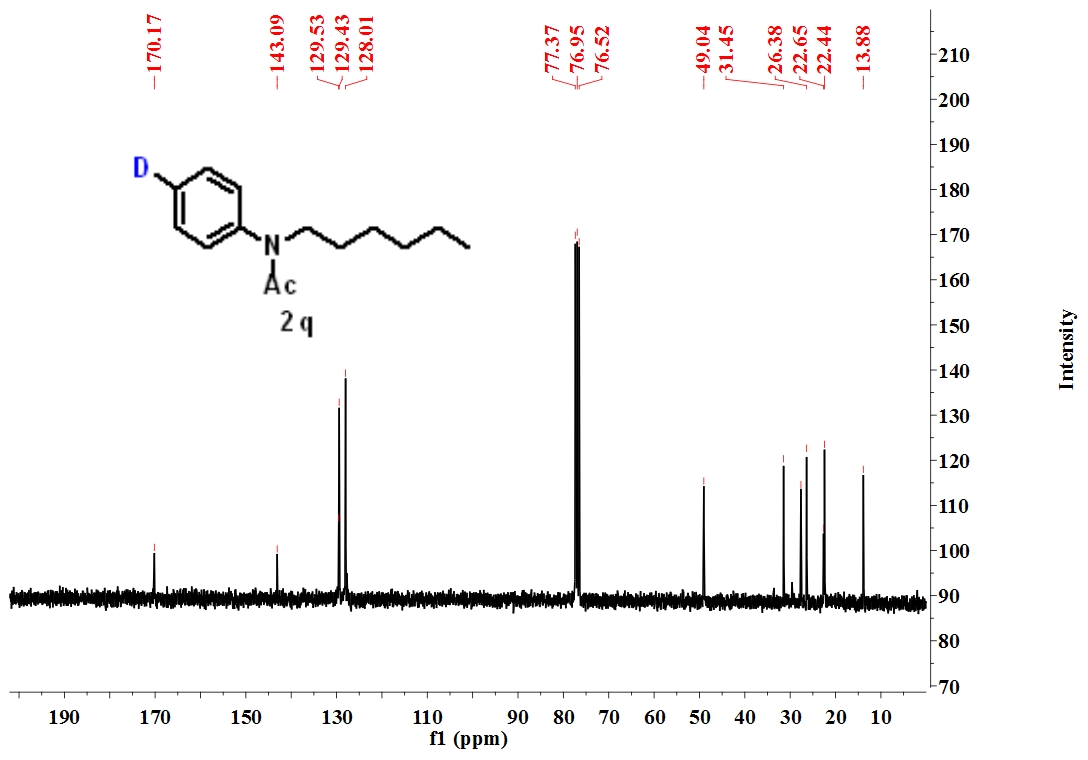


**Supplementary Figure 22.** ^13^C NMR of product **2q**. **^13^C NMR** (300 MHz, Chloroform-*d*): *δ* [ppm] 170.2, 143.1, 129.5, 129.4, 128.0, 49.0, 31.4, 27.6, 26.4, 22.6, 22.4, 13.9.


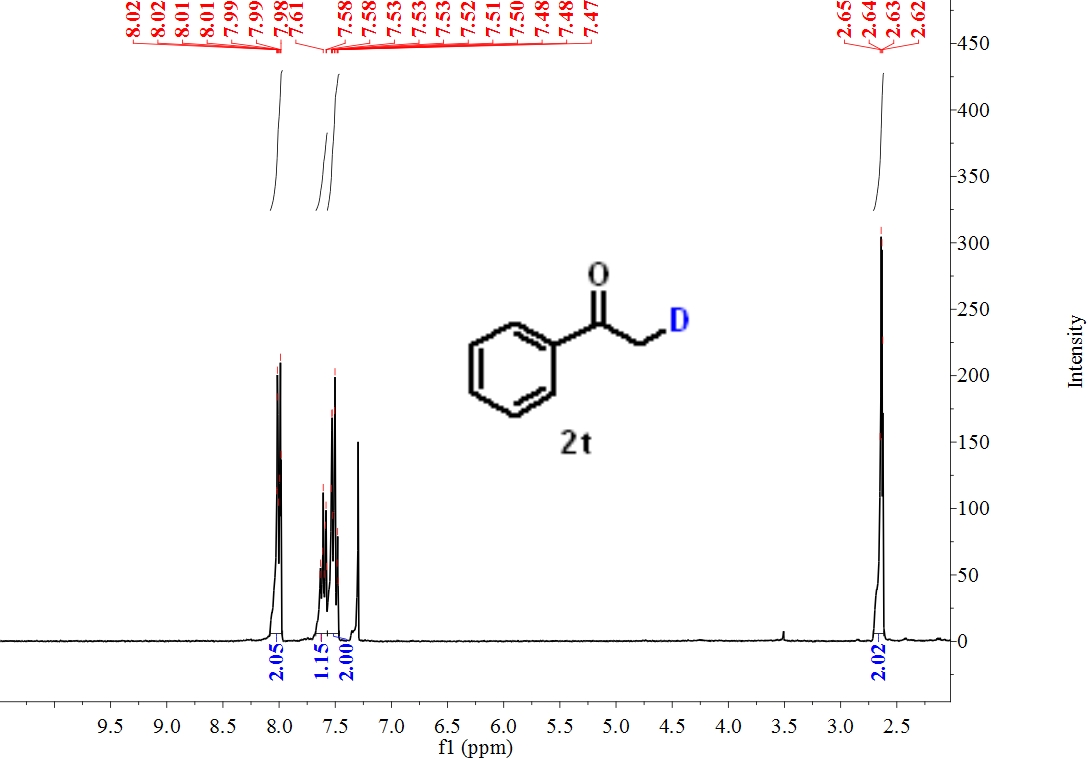


**Supplementary Figure 23.** ^1^H NMR of product **2t**. Deuterated ratio = 98%, **^1^H NMR** (300 MHz, Chloroform-*d*): *δ* [ppm] 8.08 - 7.97 (m, 2H), 7.67 - 7.57 (m, 1H), 7.57 - 7.46 (m, 2H), 2.71 - 2.62 (m, 2.02H); **HRMS** (APCI) 121.0645, theoretical value for C_8_H_7_DO 121.0638.


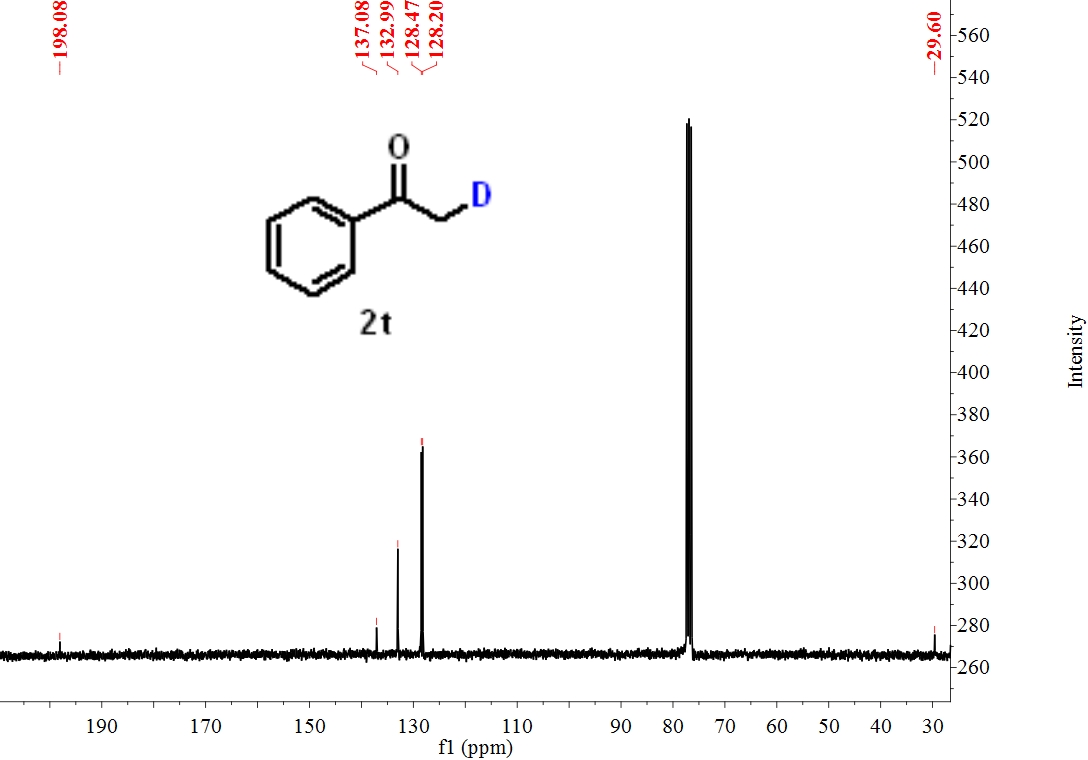


**Supplementary Figure 24.** ^13^C NMR of product **2t**. **^13^C NMR** (300 MHz, Chloroform-*d*) *δ* [ppm] 198.1, 137.1, 133.0, 128.5, 128.2, 29.6.


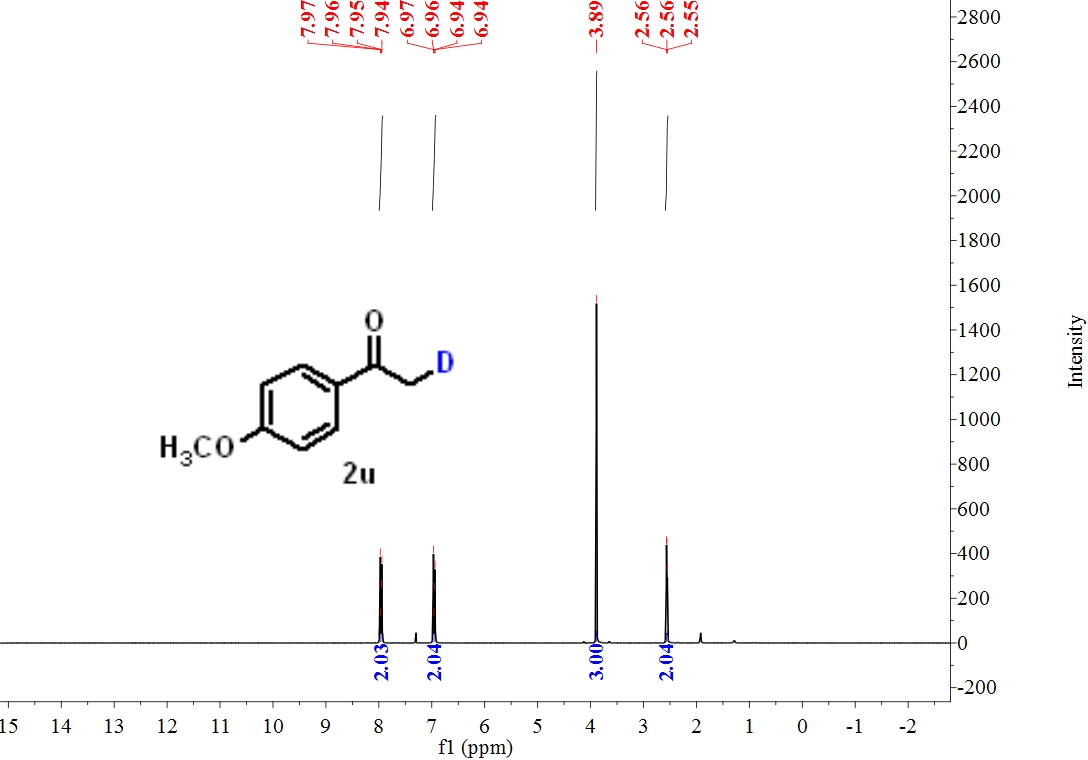


**Supplementary Figure 25.** ^1^H NMR of product **2u**. Deuterated ratio = 96%, **^1^H NMR** (300 MHz, Chloroform-*d*) *δ* [ppm] 7.99 - 7.93 (m, 2H), 6.99 - 6.92 (m, 2H), 3.89 (s, 3H), 2.59 - 2.54 (m, 2.04H); **GCMS** (EI) 151.1, theoretical value for C_9_H_9_DO_2_ 151.2.


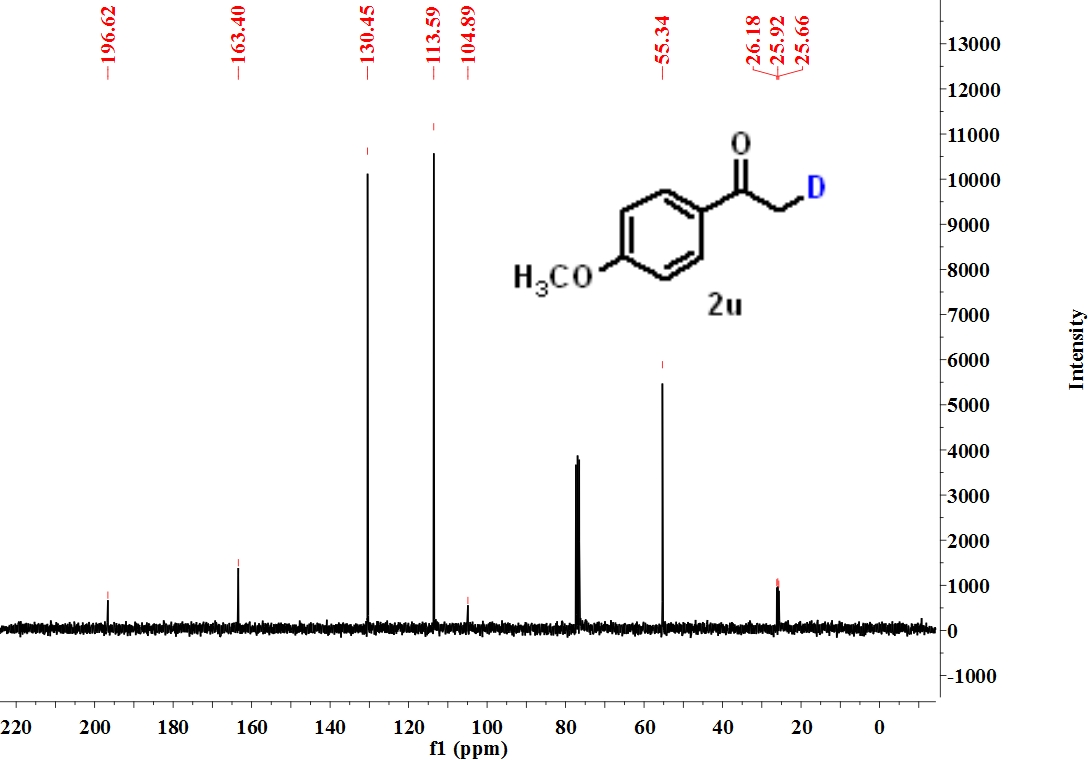


**Supplementary Figure 26.** ^13^C NMR of product **2u**. **^13^C NMR** (300 MHz, Chloroform-*d*) *δ* [ppm] 196.6, 163.4, 130.4, 113.6, 104.9, 55.3, 25.9 (t, *J* = 78 Hz).


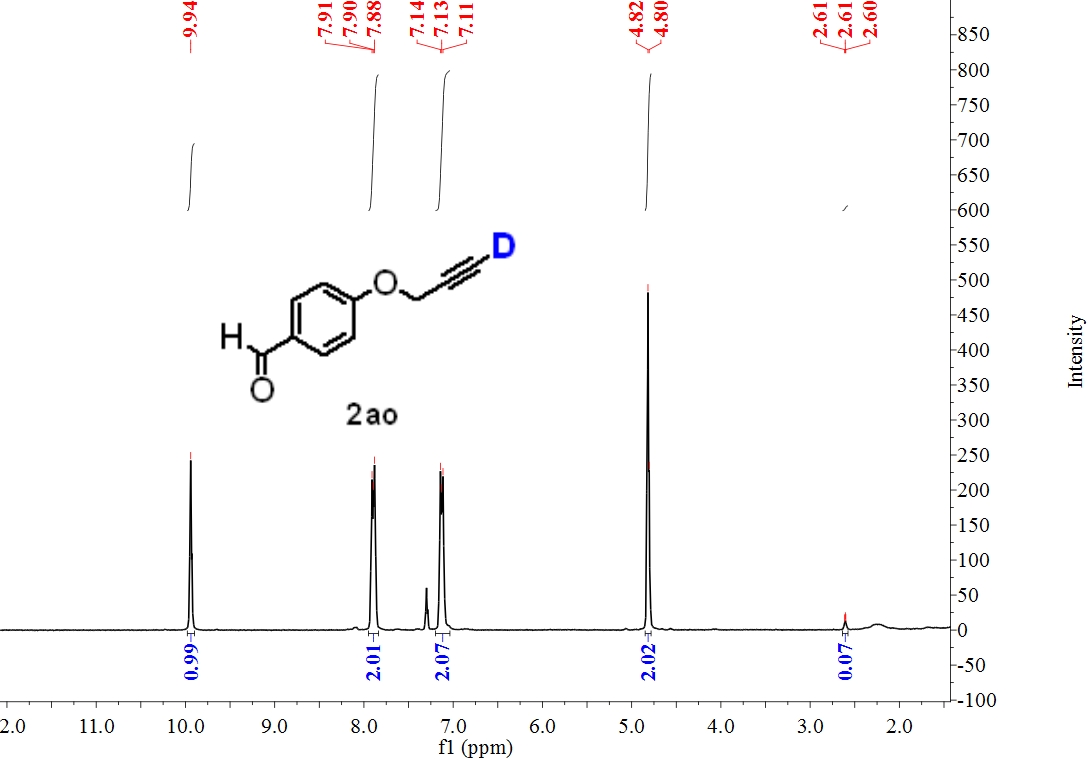


**Supplementary Figure 27.** ^1^H NMR of product **2ao**. Deuterated ratio = 93%, **^1^H NMR** (300 MHz, Chloroform-*d*): *δ* [ppm] 9.94 (s, 1H), 7.95 - 7.84 (m, 2H), 7.18 - 7.07 (m, 2H), 4.81 (d, *J* = 4.1 Hz, 2H), 2.64 - 2.58 (m, 0.07H); **HRMS** (APCI) 161.0595, theoretical value for C_10_H_7_DO_2_ 161.0587.


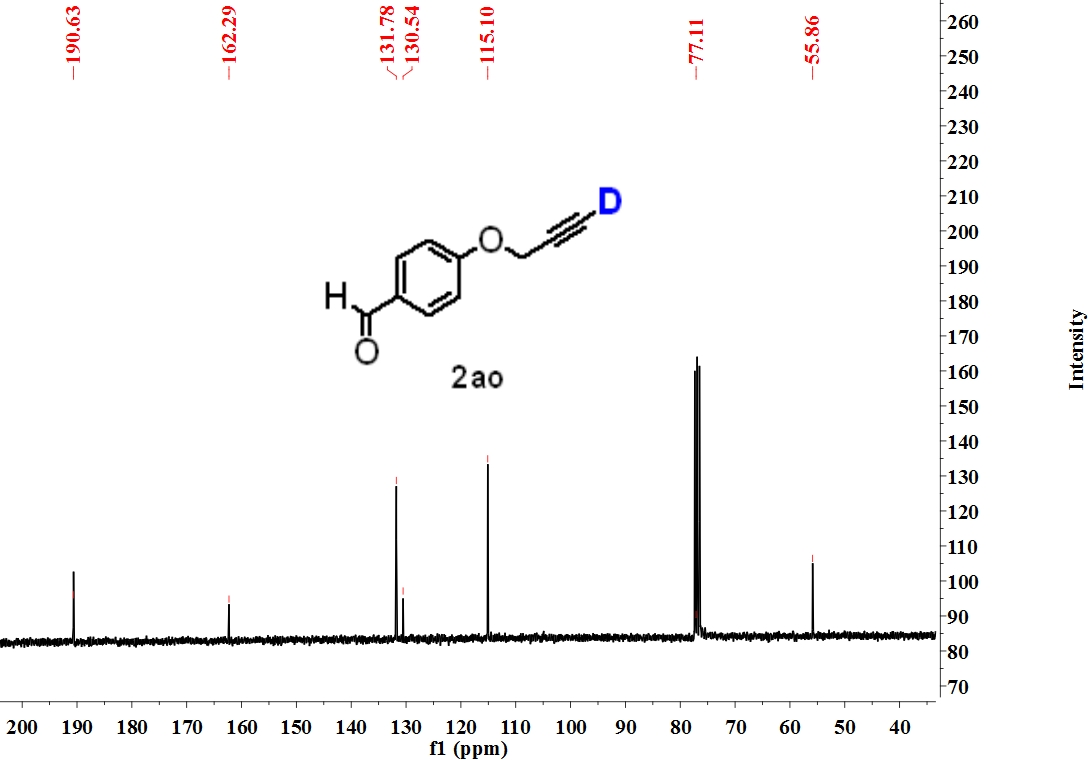


**Supplementary Figure 28.** ^13^C NMR of product **2ao**. **^13^C NMR** (300 MHz, Chloroform-*d*) *δ* [ppm] 190.6, 162.3, 131.8, 130.5, 115.1, 77.1, 55.9.


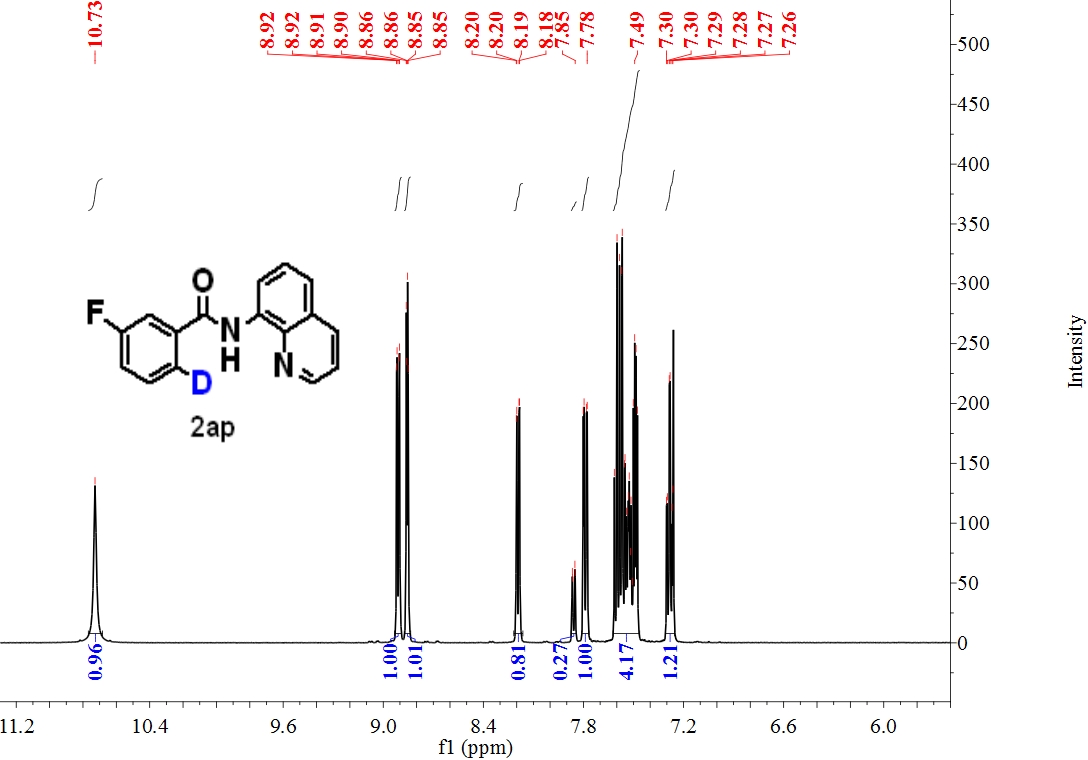


**Supplementary Figure 29.** ^1^H NMR of product **2ap**. Deuterated ratio = 73%, **^1^H NMR** (500 MHz, Chloroform-*d*) *δ* [ppm] 10.73 (s, 1H), 8.91 (dd, *J* = 7.4, 1.5 Hz, 1H), 8.86 (dd, *J* = 4.2, 1.6 Hz, 1H), 8.19 (dd, *J* = 8.3, 1.6 Hz, 1H), 7.87 - 7.84 (m, 0.27H), 7.79 (dd, *J* = 9.4, 2.6 Hz, 1H), 7.62 - 7.46 (m, 4H), 7.28 (td, *J* = 8.3, 2.6 Hz, 1H); **GCMS** (EI) 267.1, theoretical value for C_16_H_10_DFN_2_O 267.3.


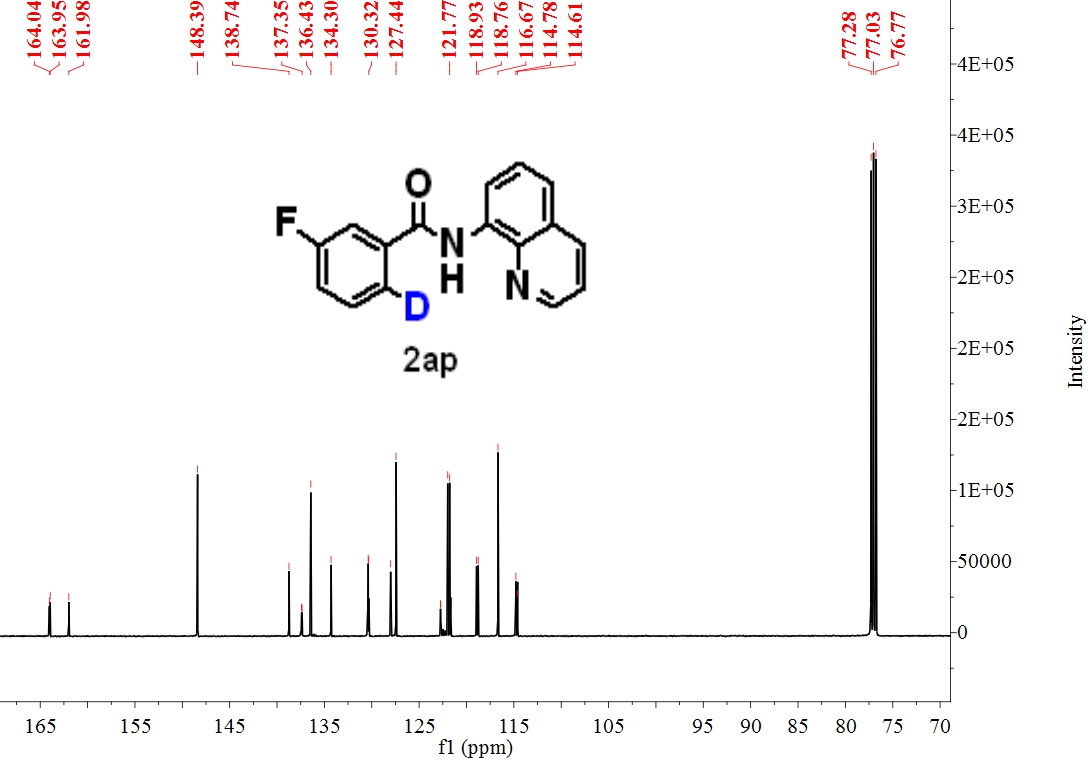


**Supplementary Figure 30.** ^13^C NMR of product **2ap**. **^13^C NMR** (500 MHz, Chloroform-*d*) *δ* [ppm] 164.0, 163.0 (d, *J^1^*_F_ = 197 Hz), 148.4, 138.7, 137.4 (d, *J^3^*_F_ = 6 Hz), 136.4, 134.3, 130.3 (d, *J^3^*_F_ = 6 Hz), 128.0, 127.4, 122.7 (d, *J^4^*_F_ = 2 Hz), 122.0, 121.8, 118.8 (d, *J^3^*_F_ = 17 Hz), 116.7, 114.7 (d, *J^3^*_F_ = 18 Hz).


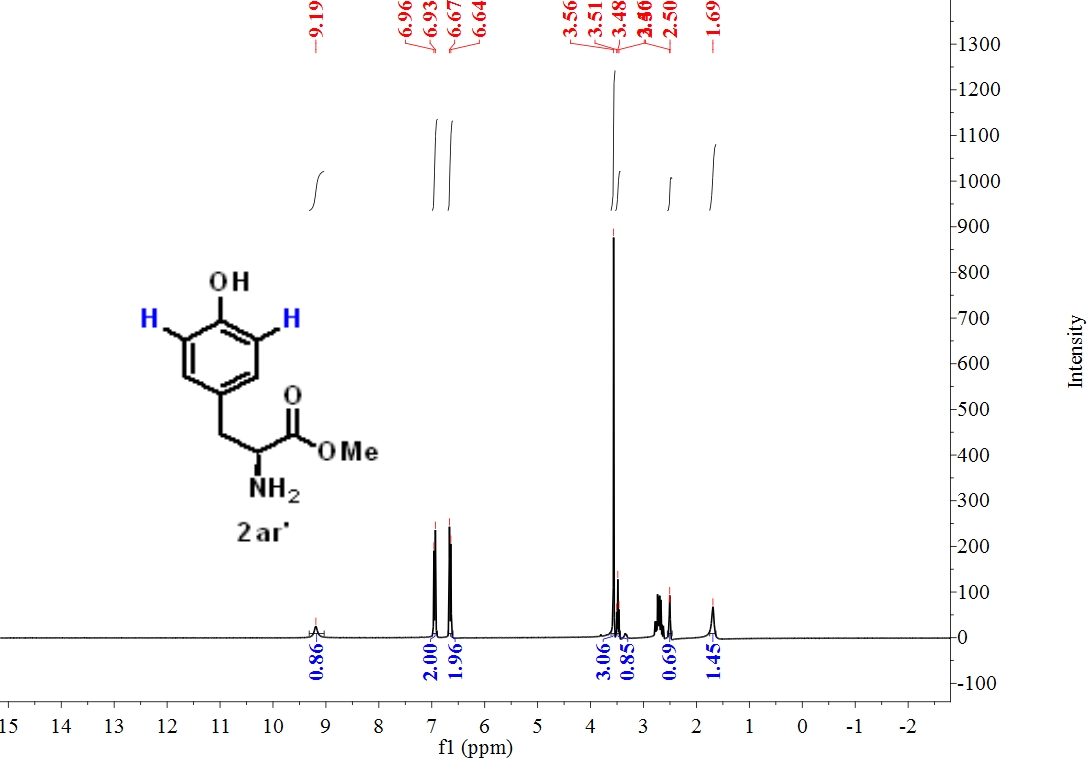


**Supplementary Figure 31.** ^1^H NMR of product **2ar’**. **^1^H NMR** (300 MHz, DMSO-*d6*): *δ* [ppm] 9.19 (s, 1H), 6.95 (d, *J* = 8.3 Hz, 2H), 6.65 (d, *J* = 8.0 Hz, 2H), 3.56 (s, 3H), 3.48 (t, *J* = 6.5 Hz, 1H), 2.51 (m, 1H), 1.69 (s, 1H); **GCMS** (EI) 195.1, theoretical value for C_10_H_13_NO_3_ 195.2.


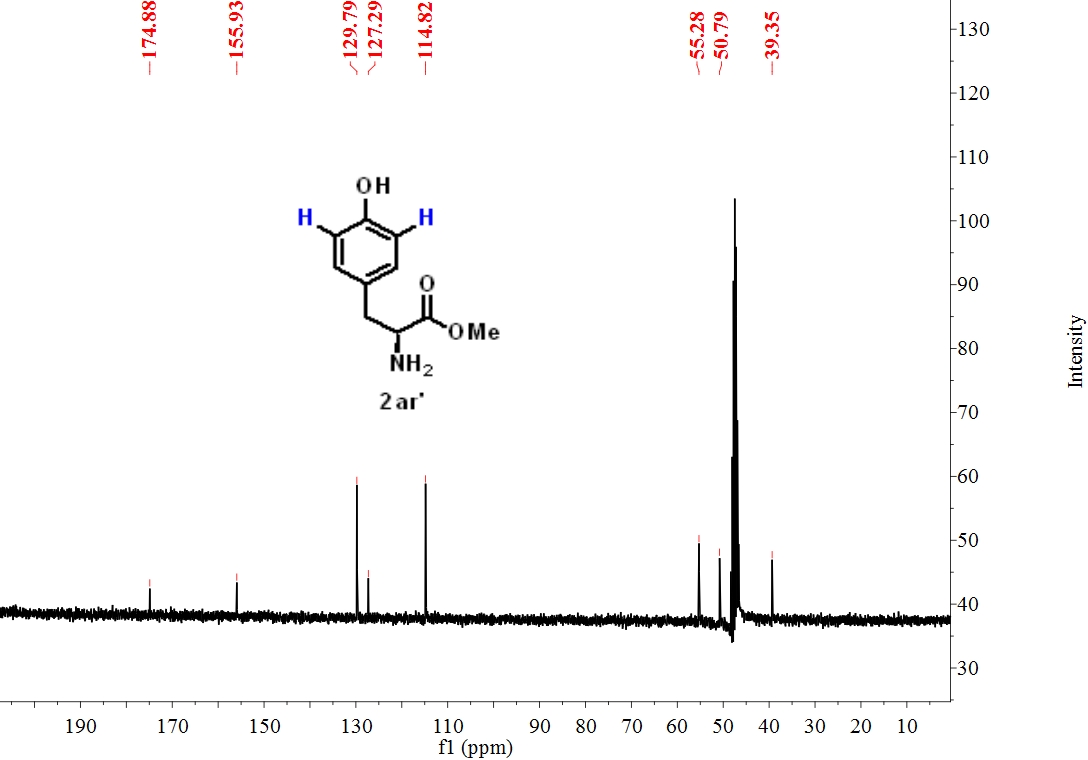


**Supplementary Figure 32.** ^13^C NMR of product **2ar’**. **^13^C NMR** (300 MHz, Methanol-*d4*): *δ* [ppm] 174.9, 155.9, 129.8, 127.3, 114.8, 55.3, 50.8, 39.4.

**
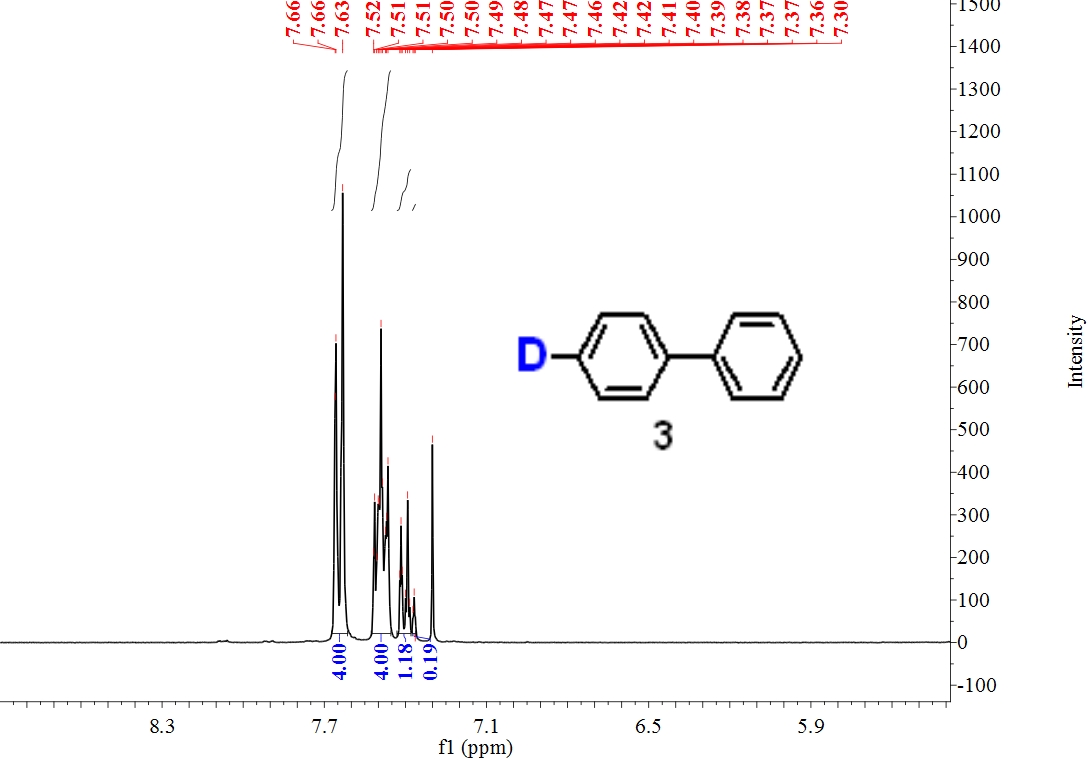
**

**Supplementary Figure 33.** ^1^H NMR of product **3**.


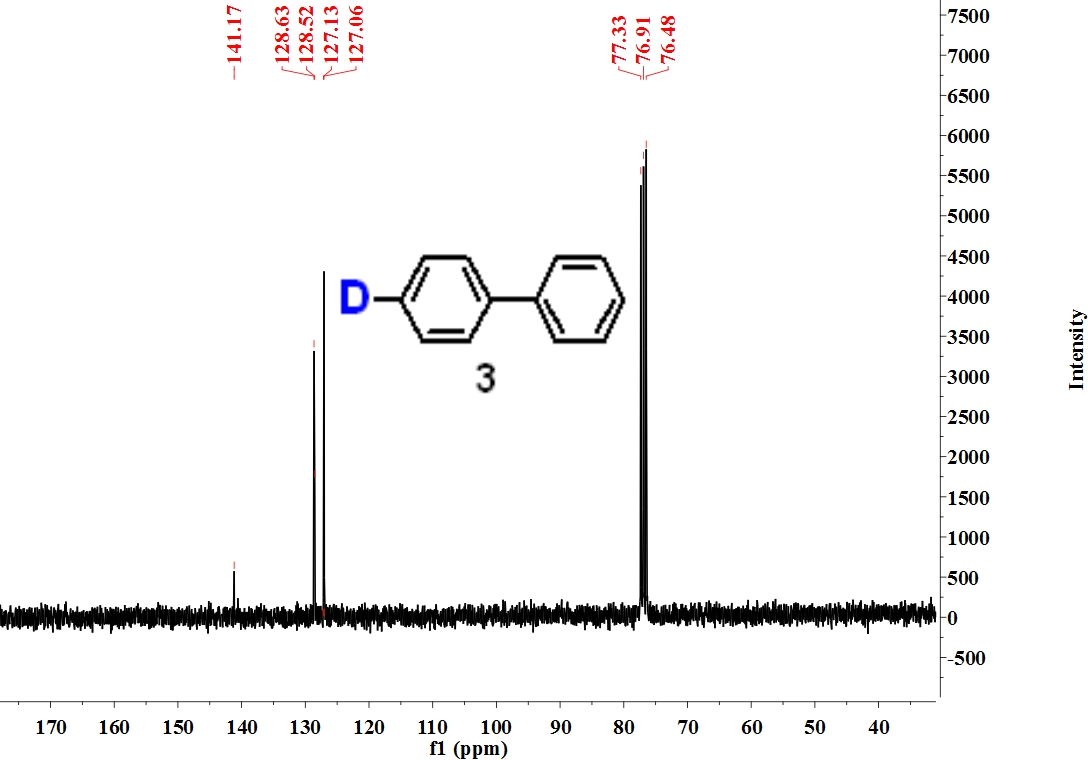


**Supplementary Figure 34.** ^13^C NMR of product **3**.


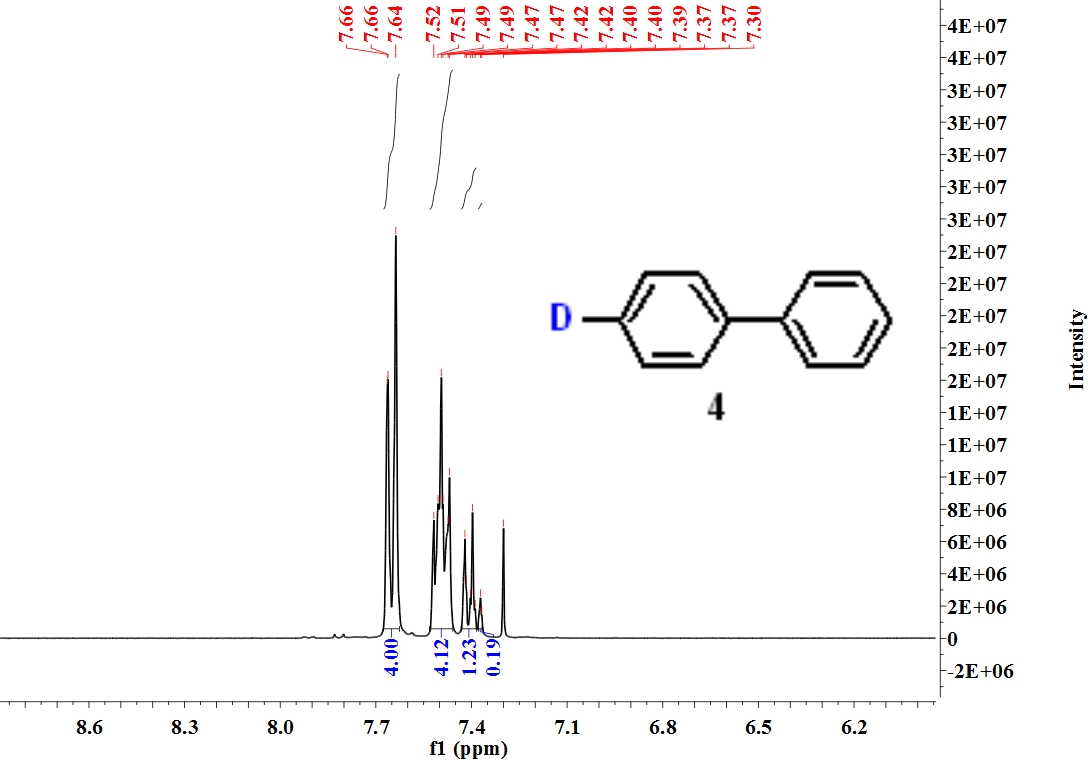


**Supplementary Figure 35.** ^1^H NMR of product **4**.


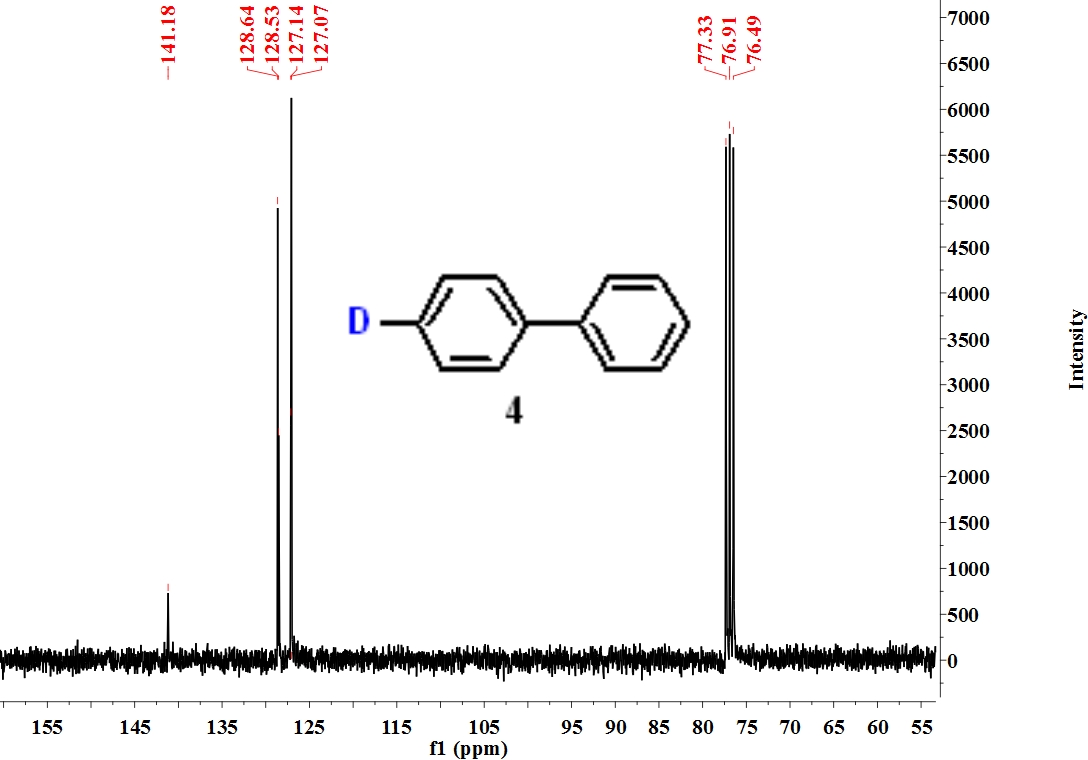


**Supplementary Figure 36.** ^13^C NMR of product **4**.

**
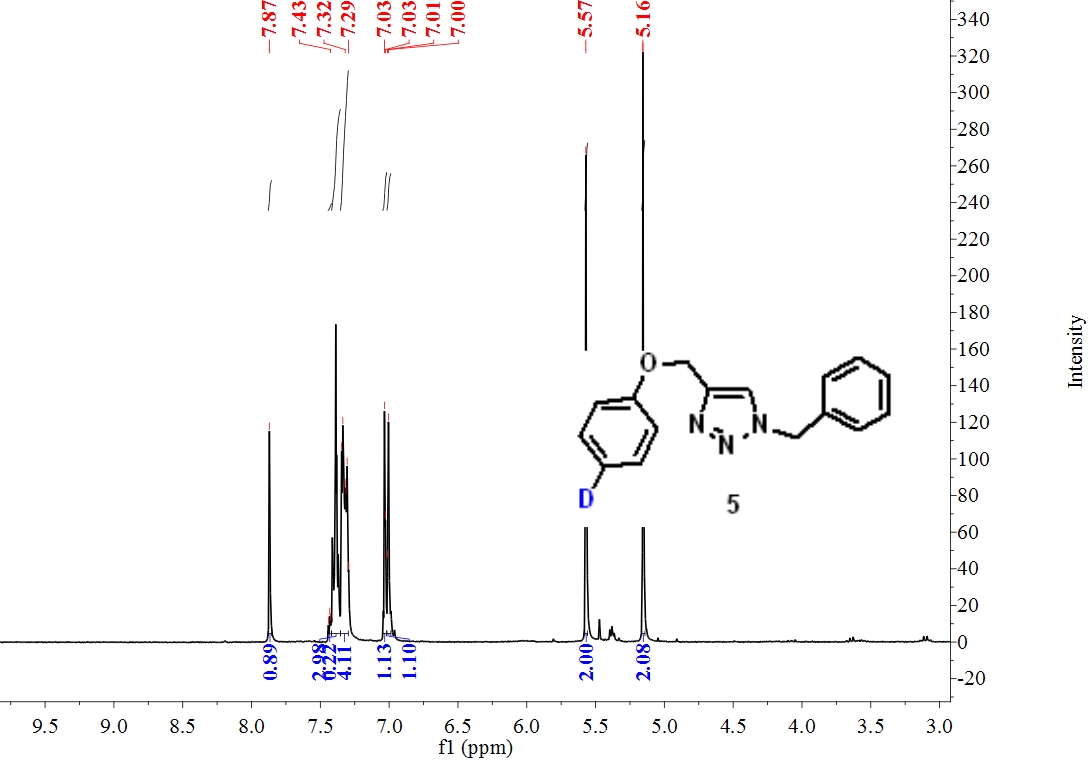
**

**Supplementary Figure 37.** ^1^H NMR of product **5**.


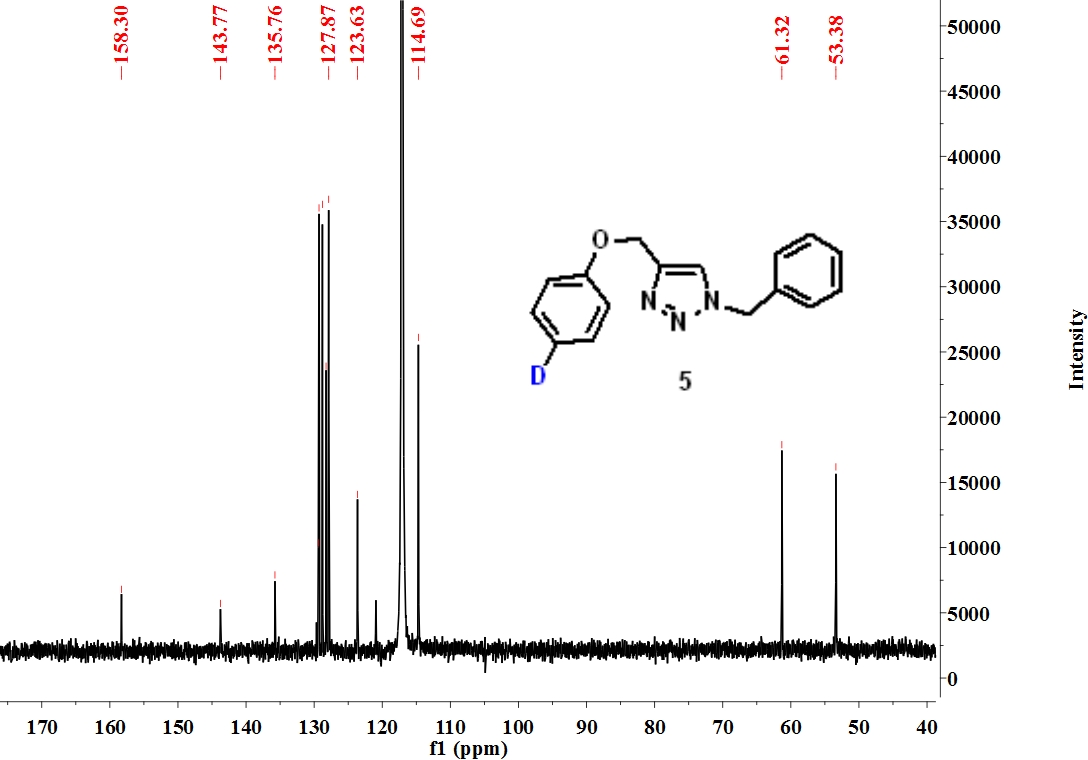


**Supplementary Figure 38.** ^13^C NMR of product **5**.

**
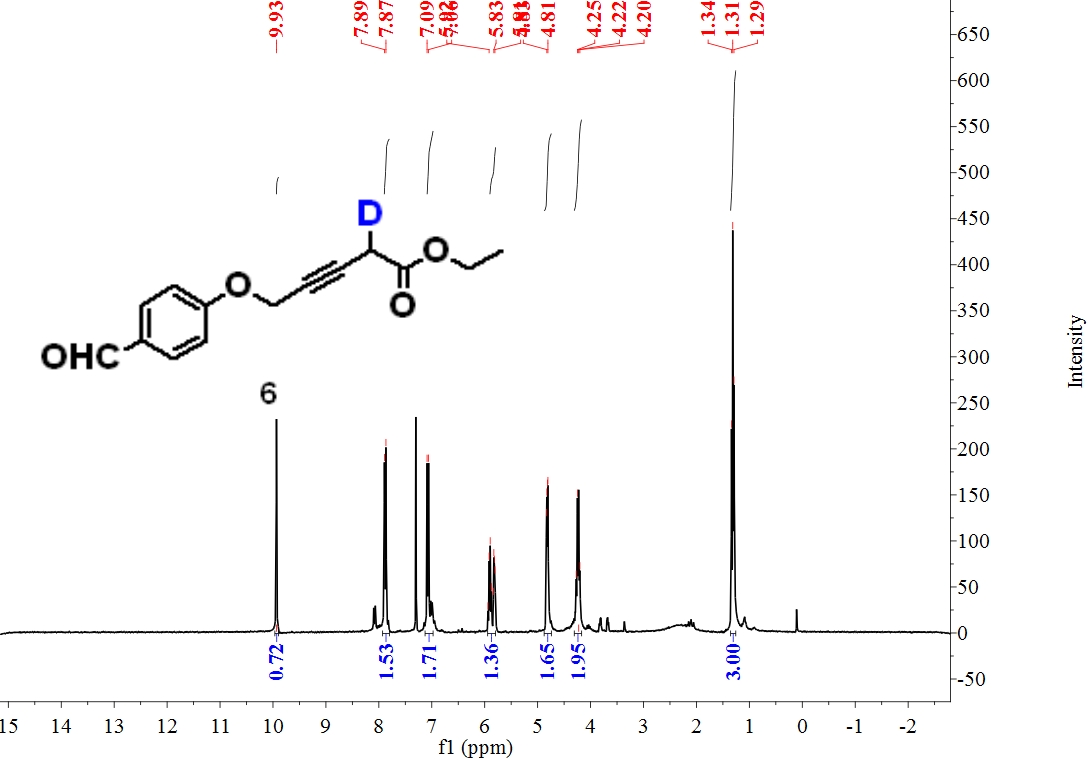
**

**Supplementary Figure 39.** ^1^H NMR of product **6**.


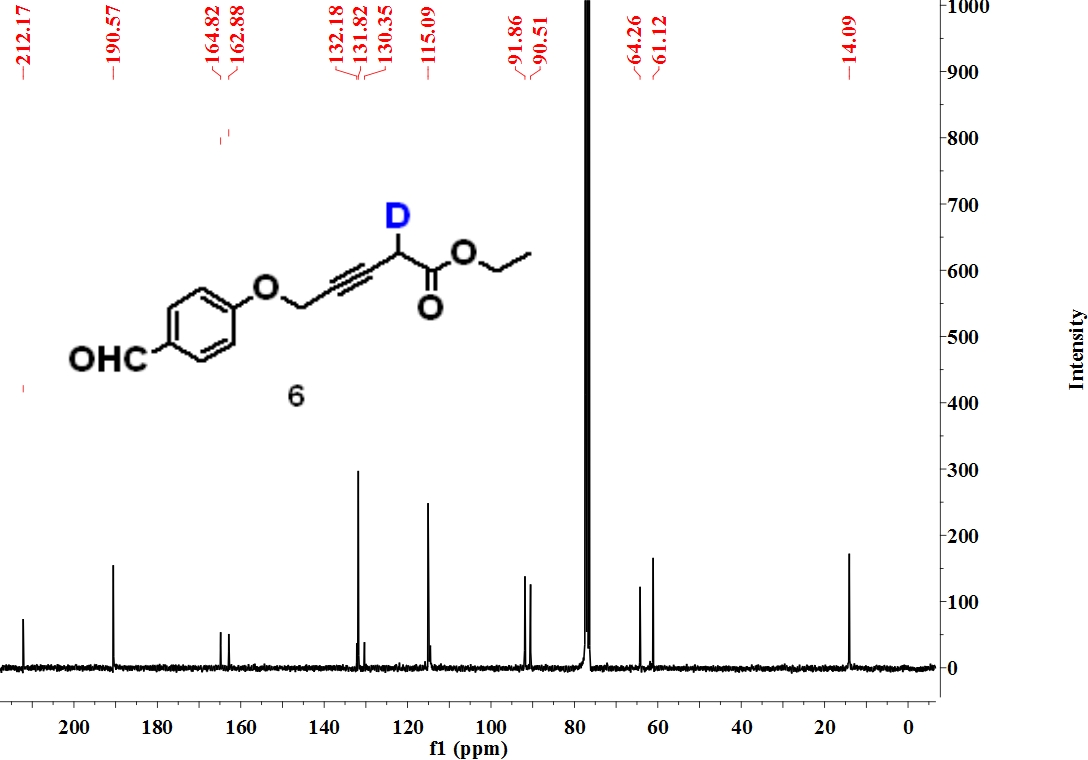


**Supplementary Figure 40.** ^13^C NMR of product **6**.

**Supplementary Figure 41.** Synthesis of substrate **7**.


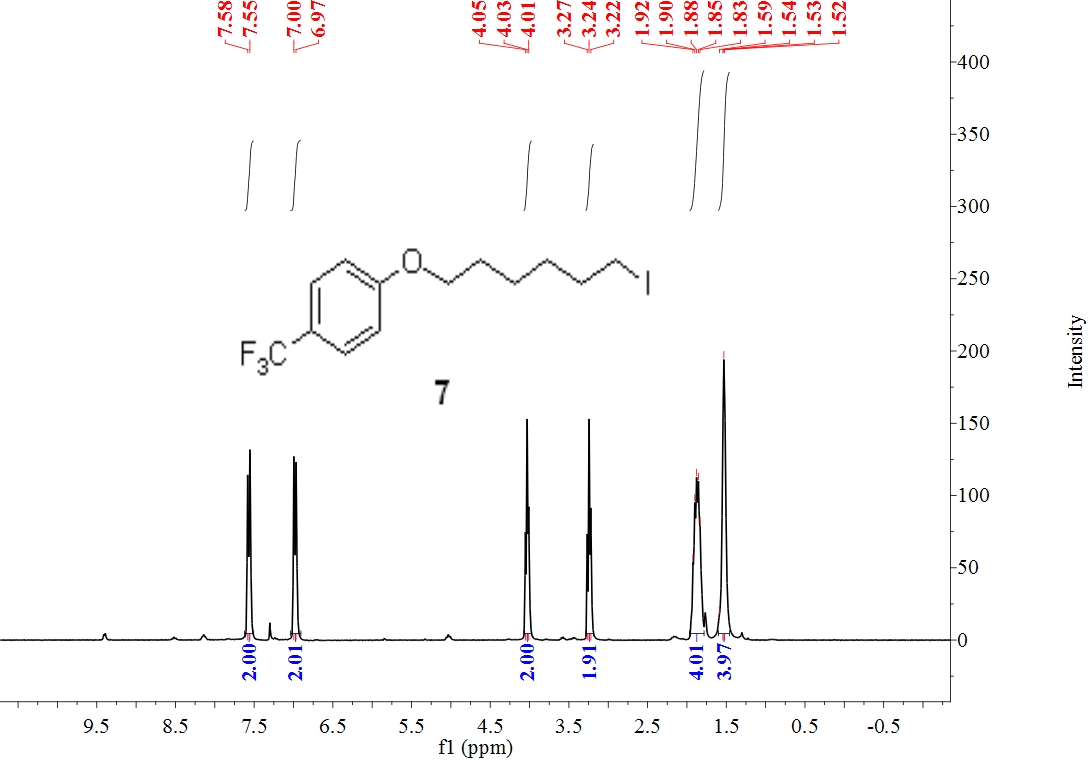


**Supplementary Figure 42.** ^1^H NMR of substrate **7**.


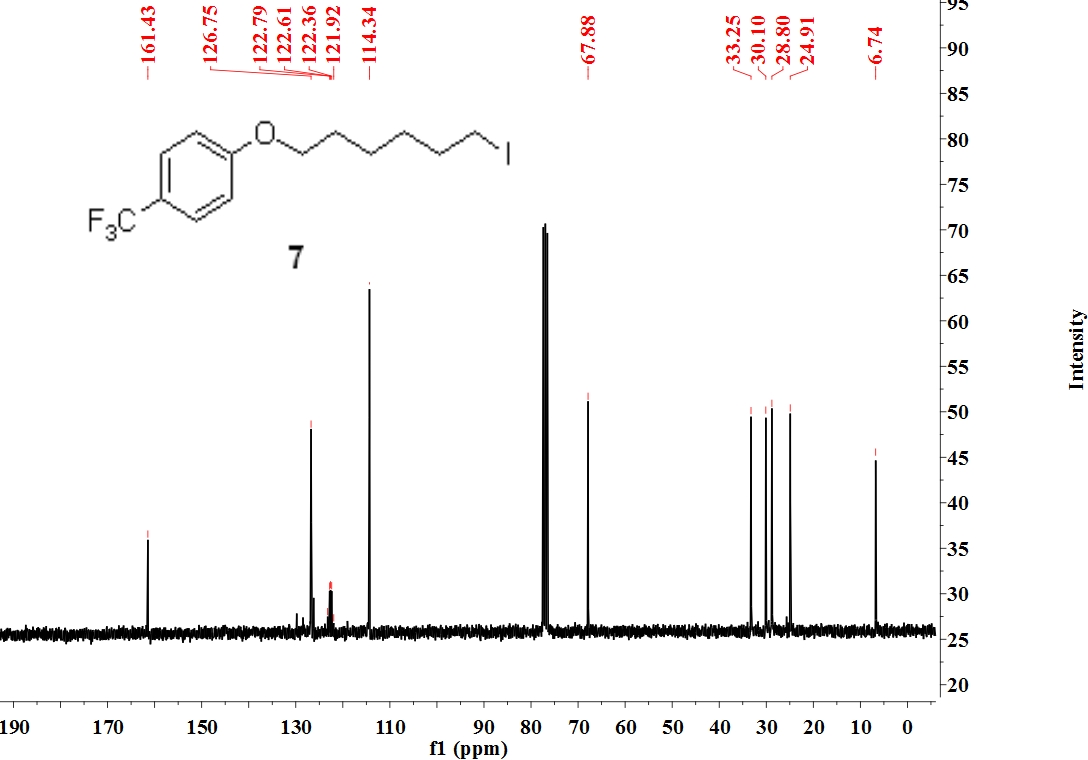


**Supplementary Figure 43.** ^13^C NMR of substrate **7**.

 **Supplementary Figure 44.** Synthesis of substrate **8**.


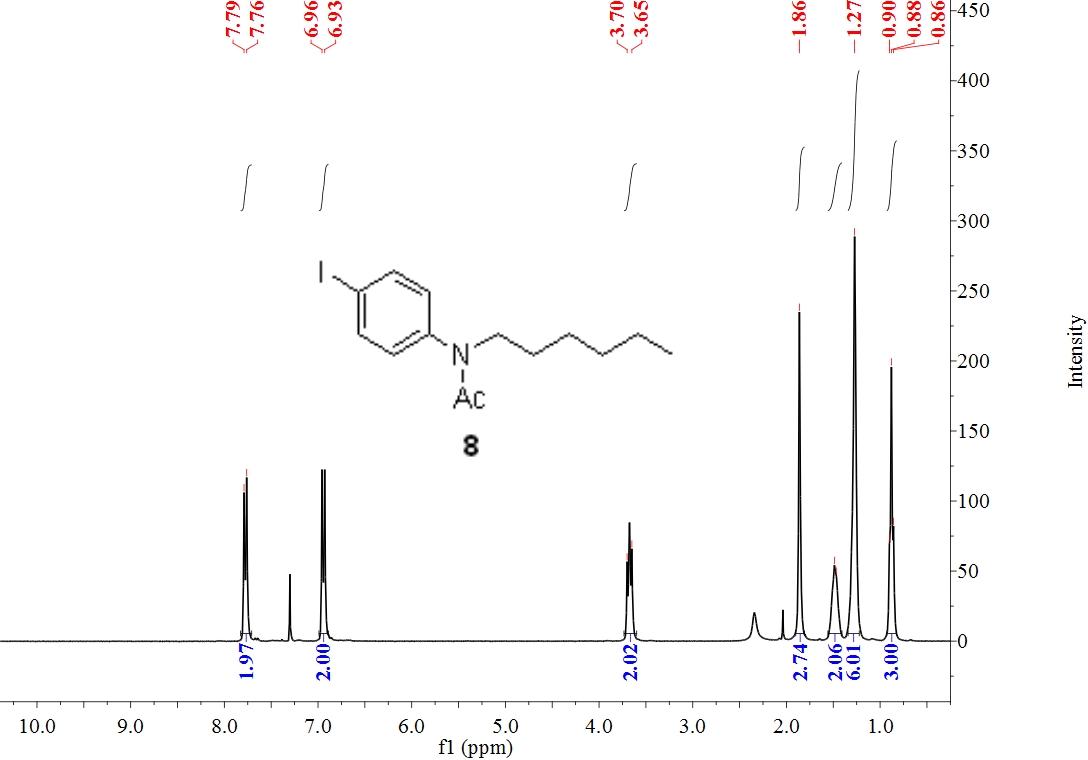


**Supplementary Figure 45.** ^1^H NMR of substrate **8**.


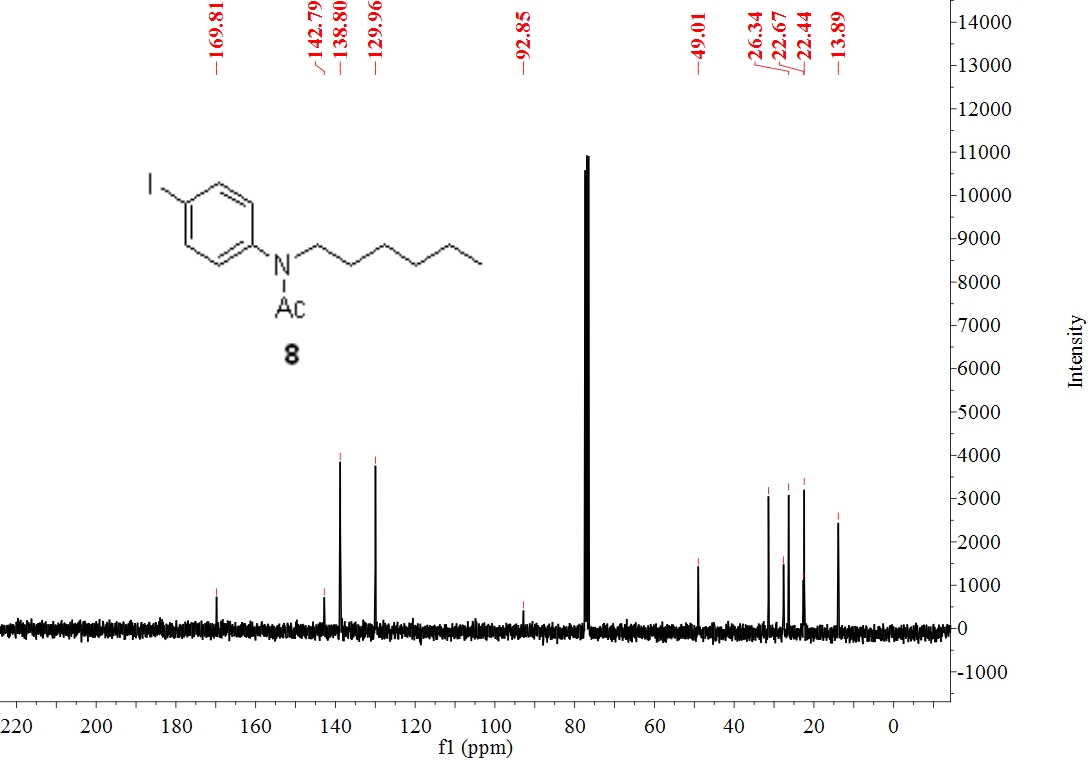


**Supplementary Figure 46.** ^13^C NMR of substrate **8**.

**Supplementary Figure 47.** Synthesis of substrate **9**.


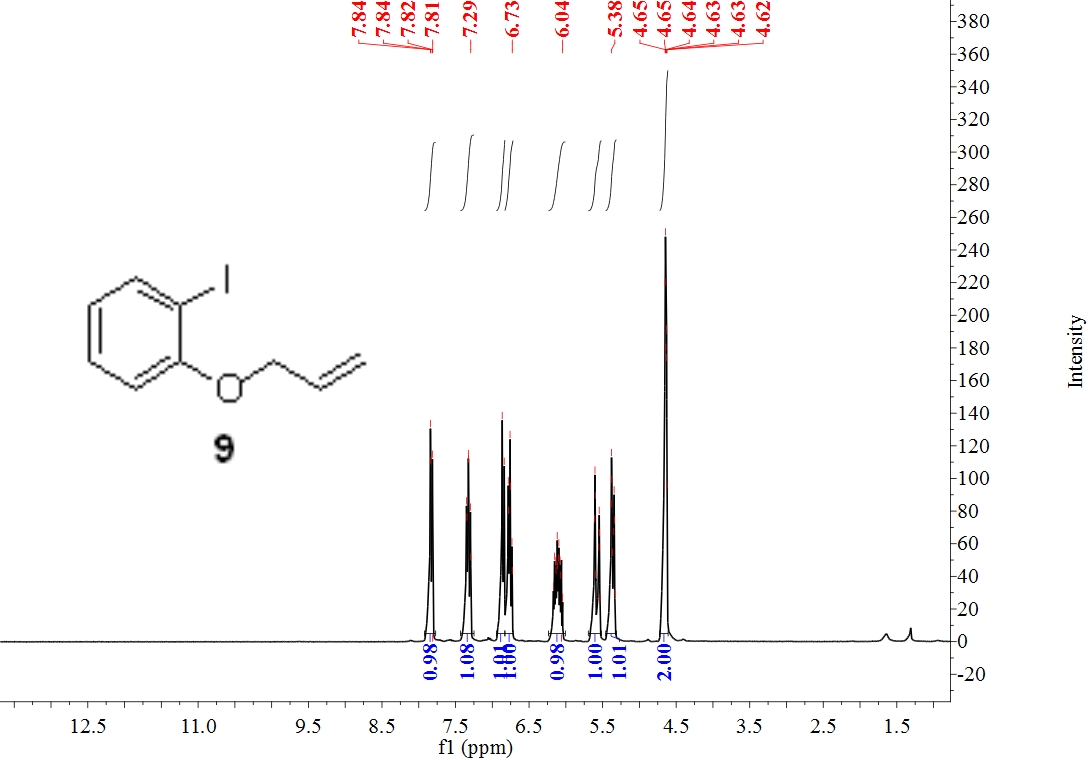


**Supplementary Figure 48.** ^1^H NMR of substrate **9**.


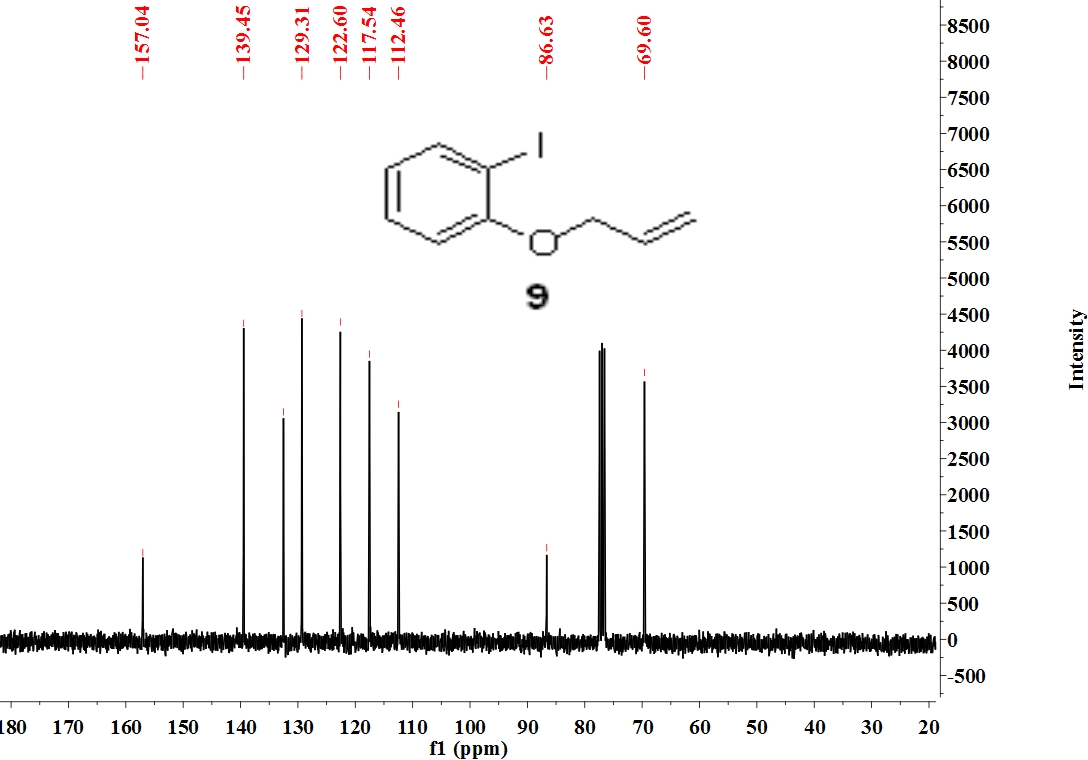


**Supplementary Figure 49.** ^13^C NMR of substrate **9**.

**Supplementary Figure 50.** Synthesis of substrate **10**.


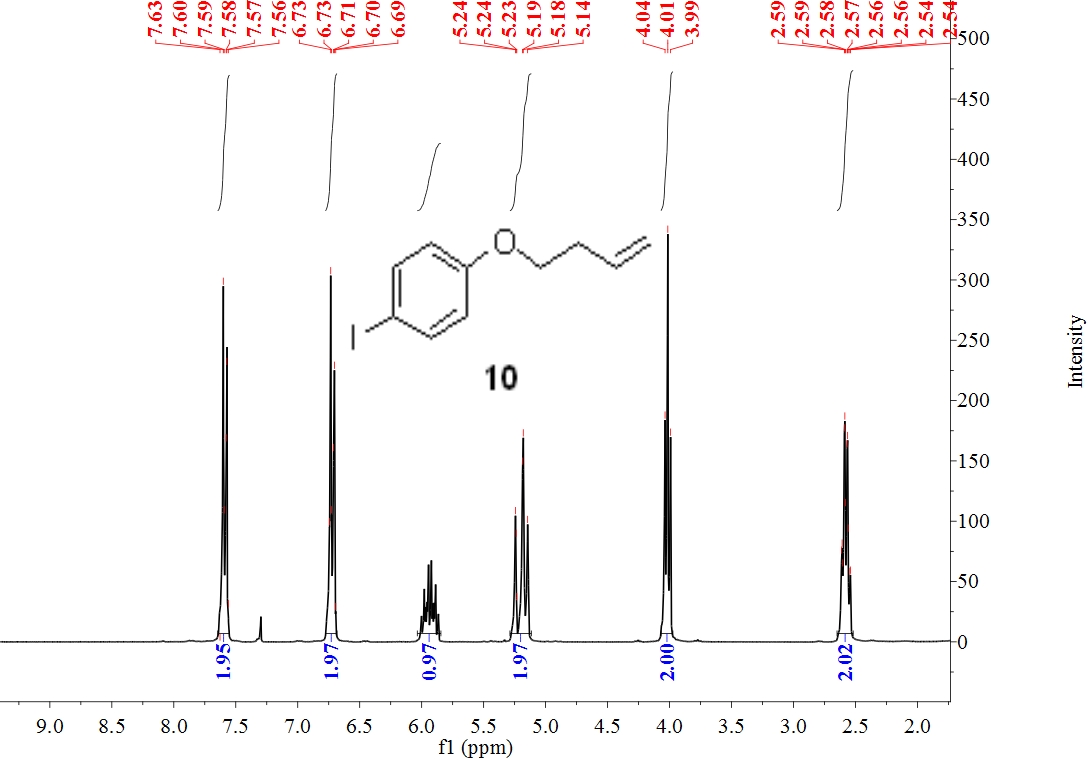


**Supplementary Figure 51.** ^1^H NMR of substrate **10**.


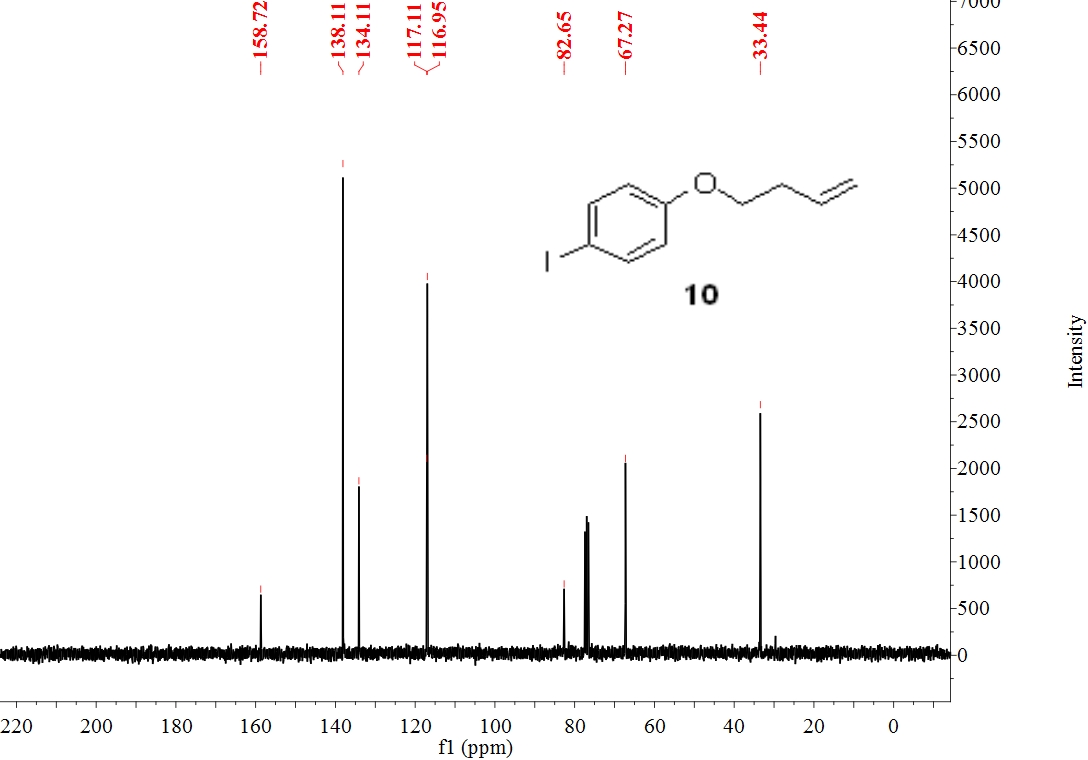


**Supplementary Figure 52.** ^13^C NMR of substrate **10**.

**Supplementary Figure 53.** Synthesis of substrate **11**.


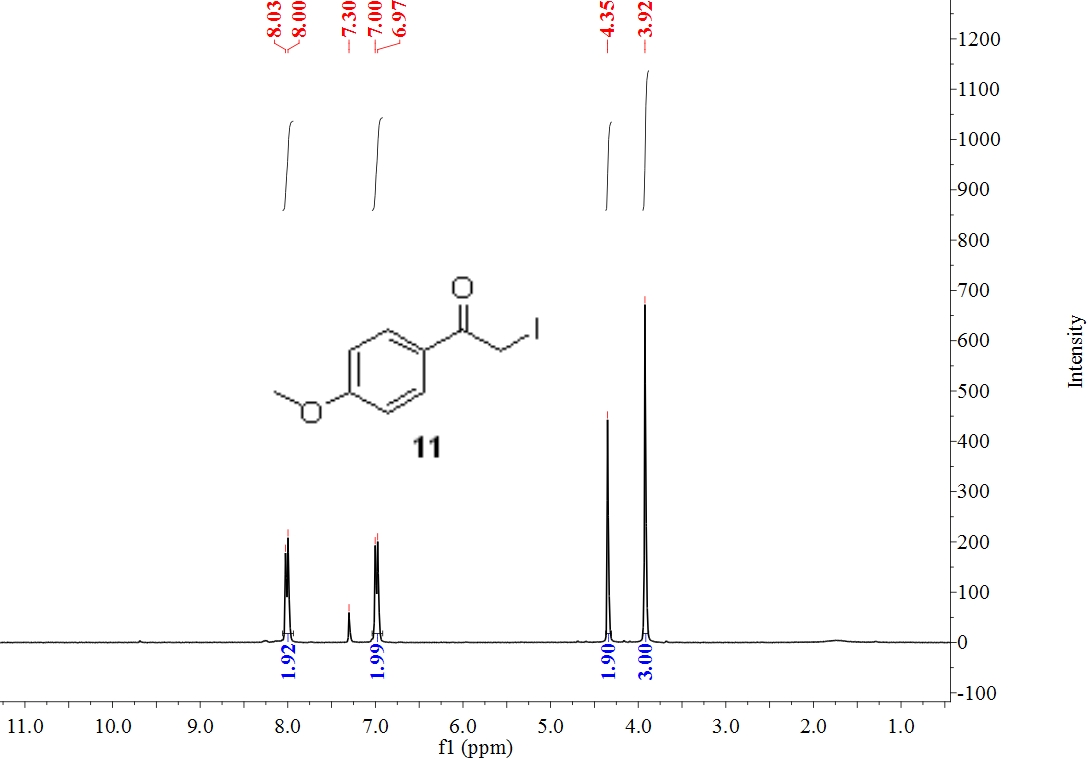


**Supplementary Figure 54.** ^1^H NMR of substrate **11**.


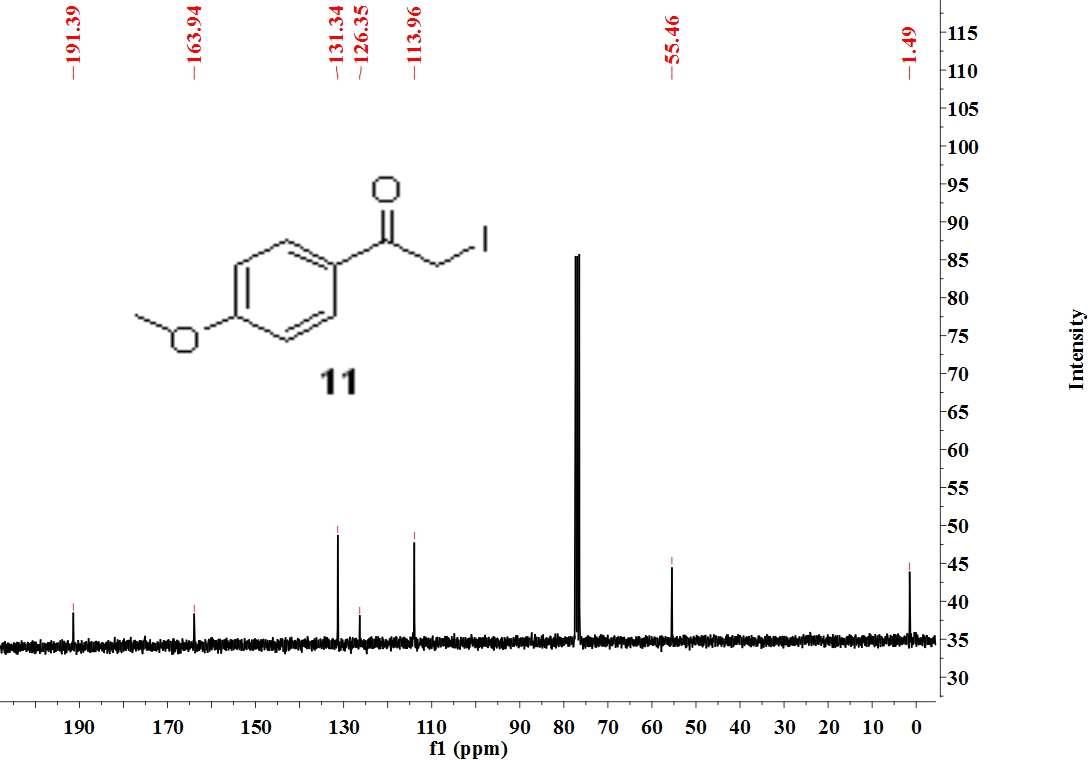


**Supplementary Figure 55.** ^13^C NMR of substrate **11**.

**Supplementary Figure 56.** Synthesis of substrate **12**.


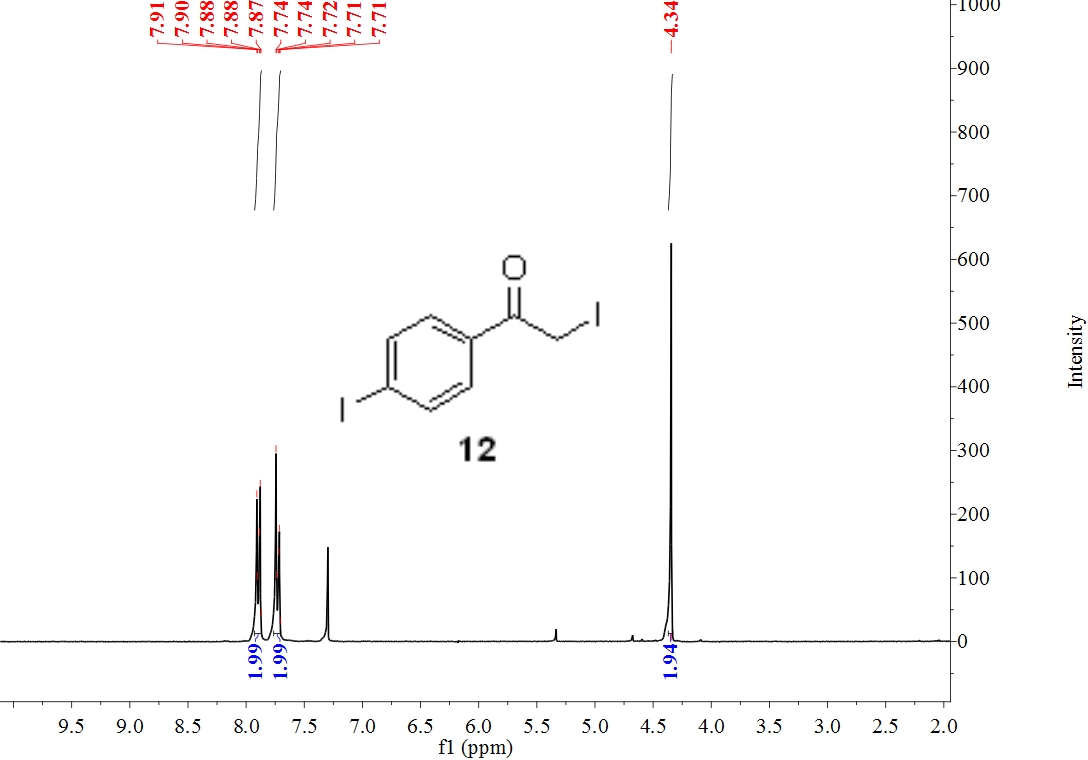


**Supplementary Figure 57.** ^1^H NMR of substrate **12**.


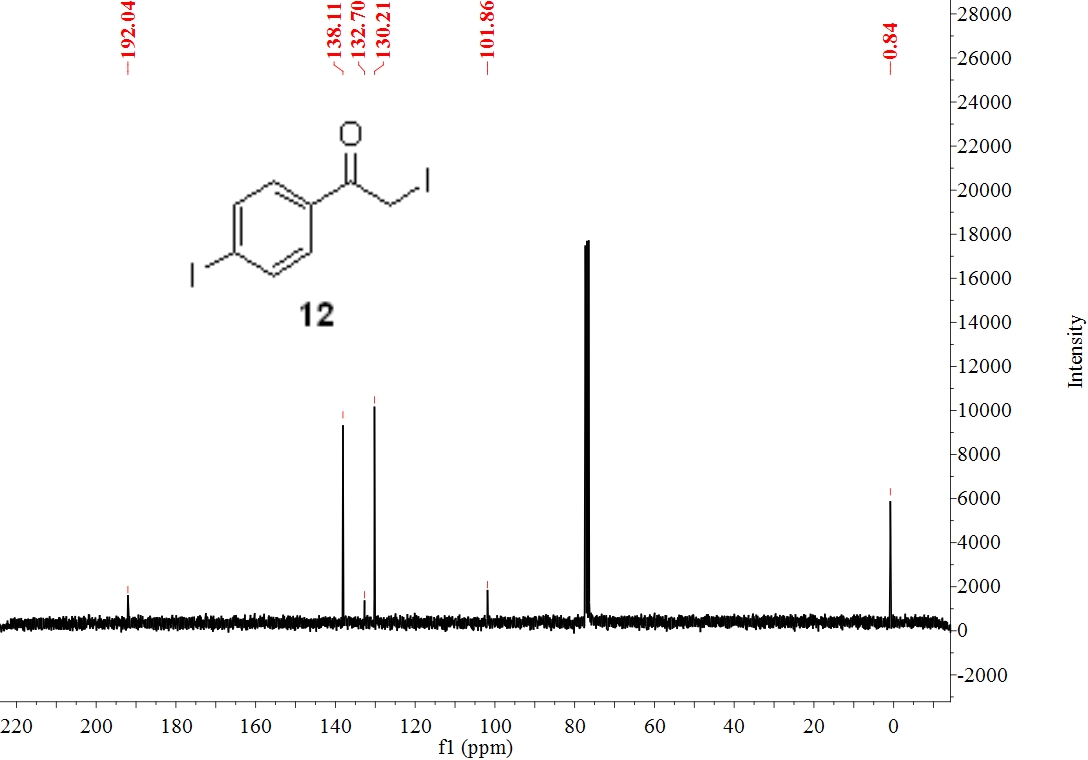


**Supplementary Figure 58.** ^13^C NMR of substrate **12**.

**Supplementary Figure 59.** Synthesis of substrate **13**.


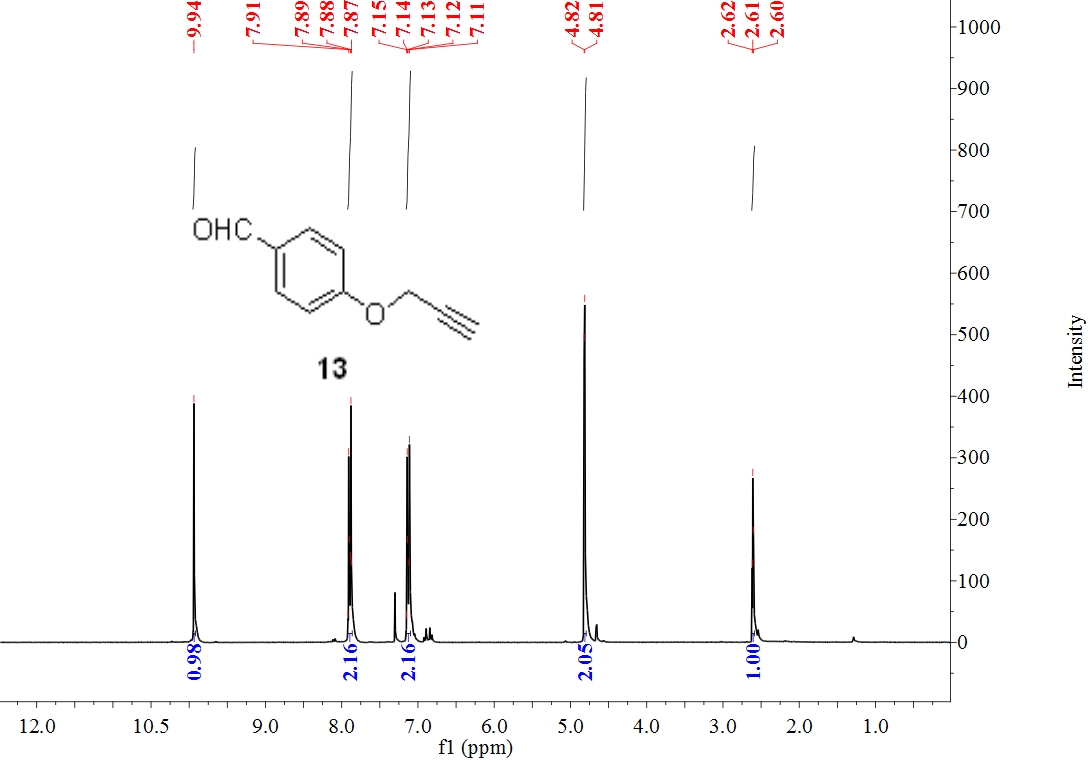


**Supplementary Figure 60.** ^1^H NMR of substrate **13**.


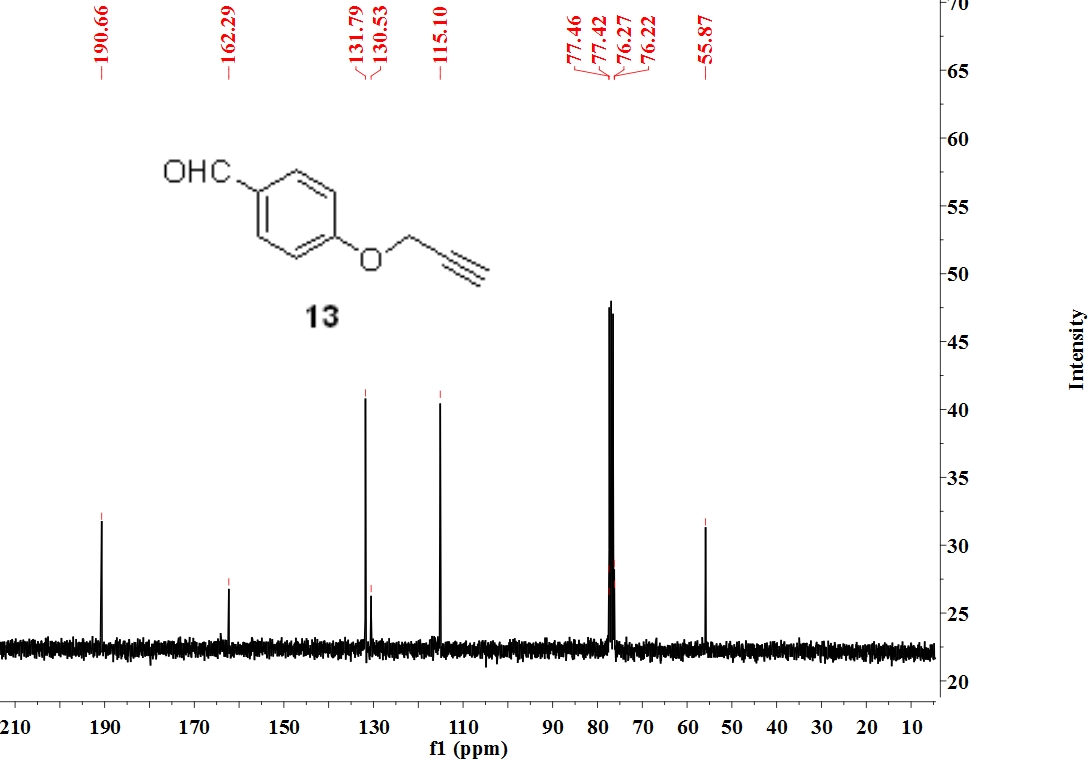


**Supplementary Figure 61.** ^13^C NMR of substrate **13**.

**Supplementary Figure 62.** Synthesis of substrate **14**.


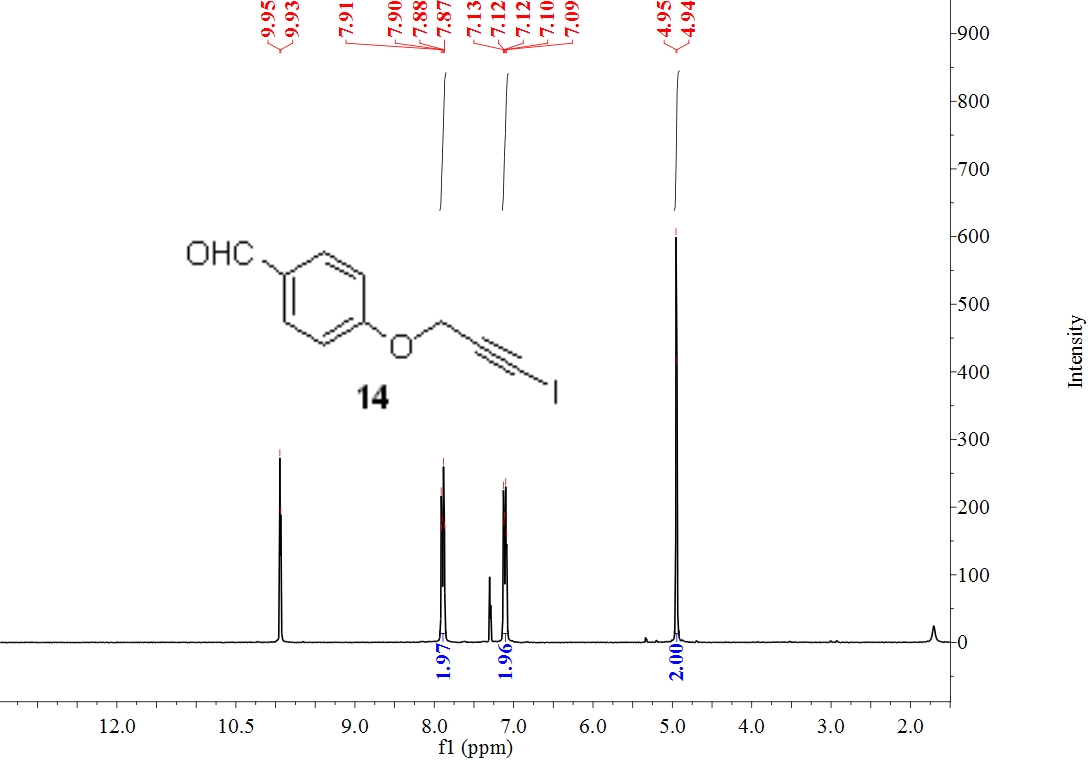


**Supplementary Figure 63.** ^1^H NMR of substrate **14**.


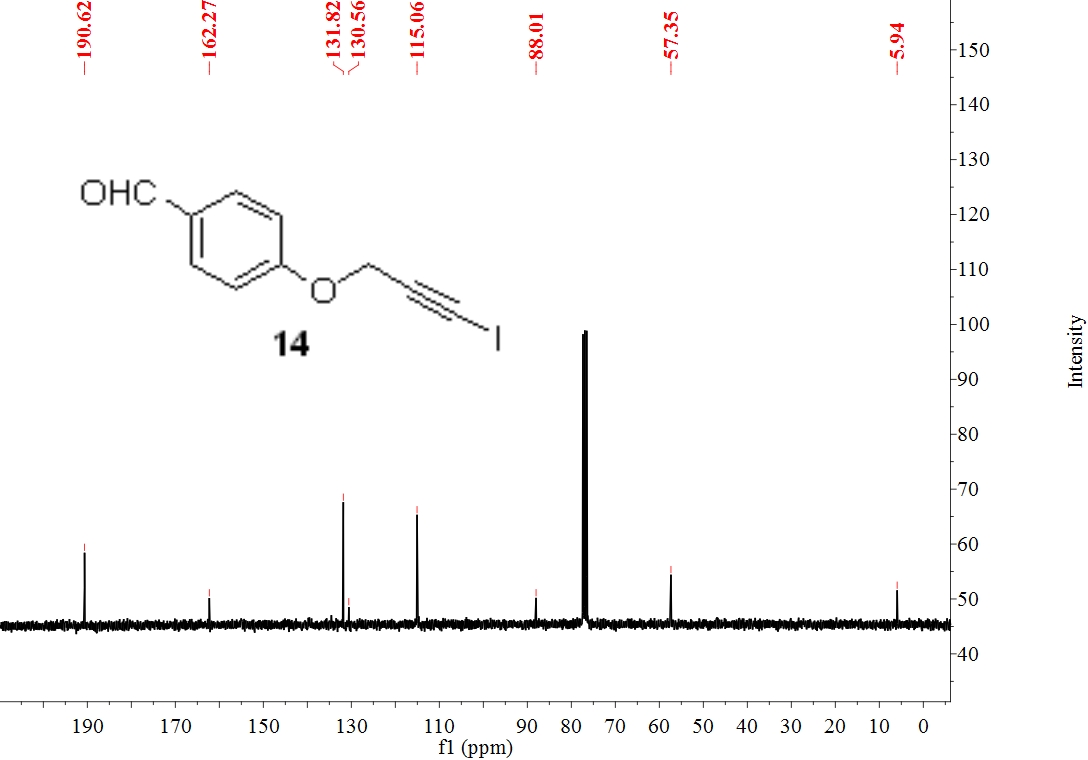


**Supplementary Figure 64.** ^13^C NMR of substrate **14**.

**Supplementary Figure 65.** Synthesis of substrate **15**.


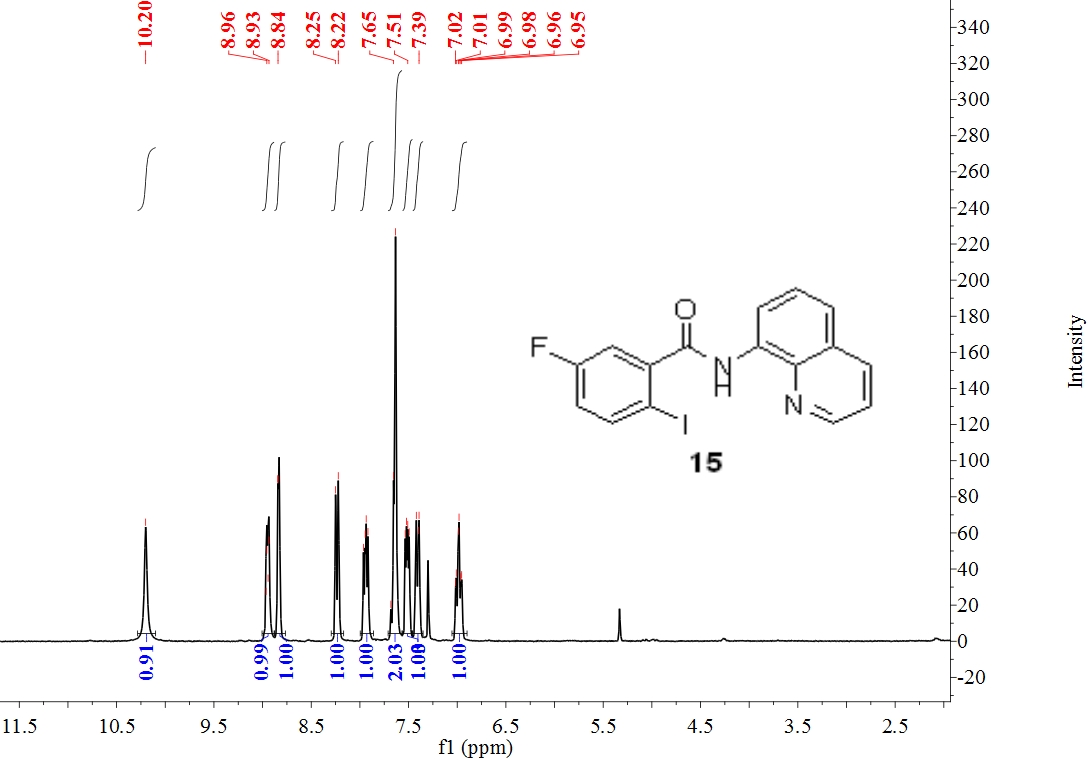


**Supplementary Figure 66.** ^1^H NMR of substrate **15**.


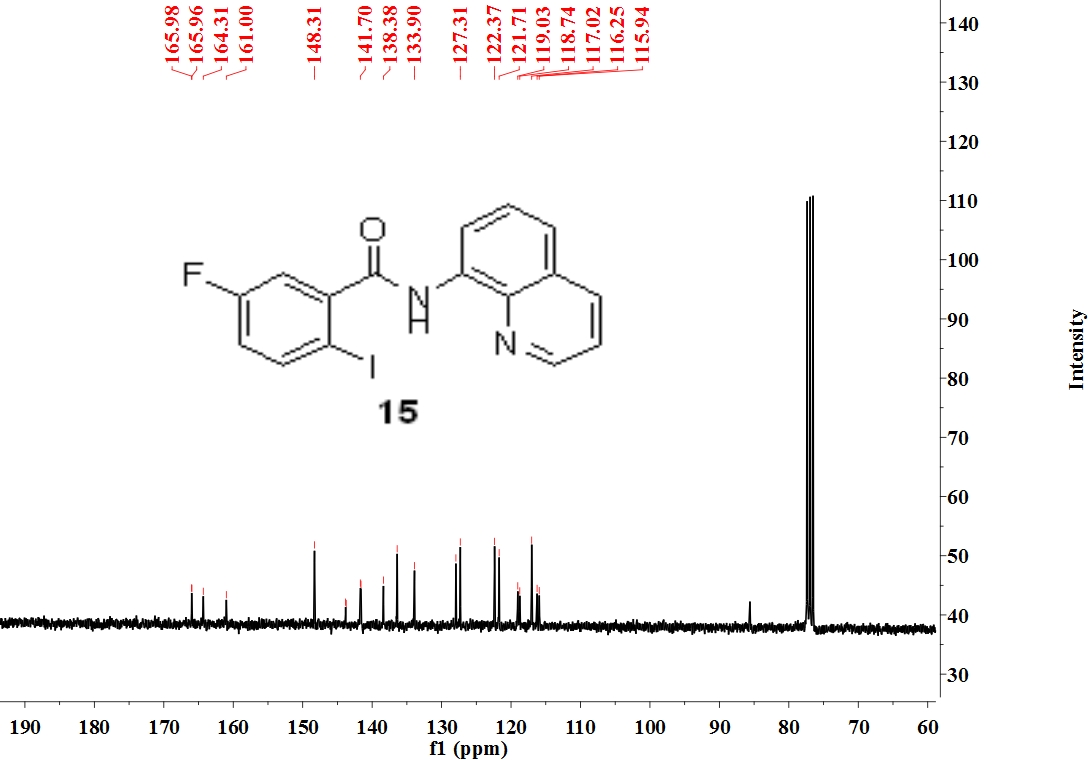


**Supplementary Figure 67.** ^13^C NMR of substrate **15**.

**Supplementary Figure 68.** Synthesis of substrate **16**.


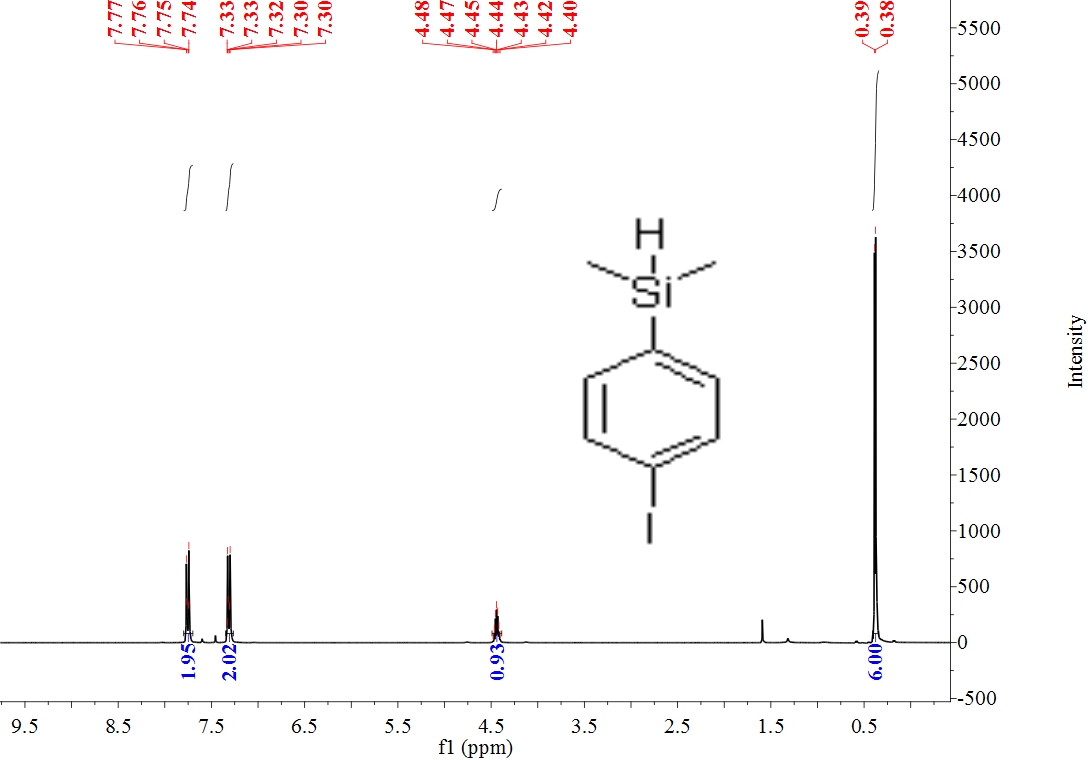


**Supplementary Figure 69.** ^1^H NMR of substrate **16**.


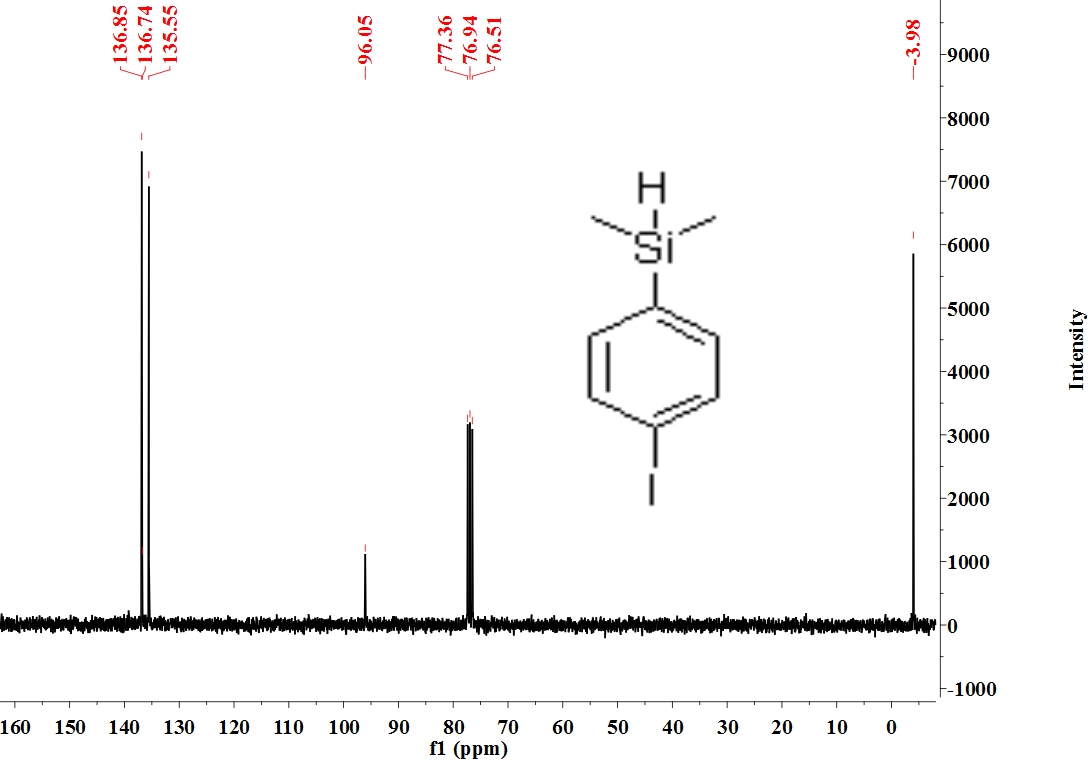


**Supplementary Figure 70.** ^13^C NMR of substrate **16**.

**Supplementary Figure 71.** Synthesis of substrate **17**.


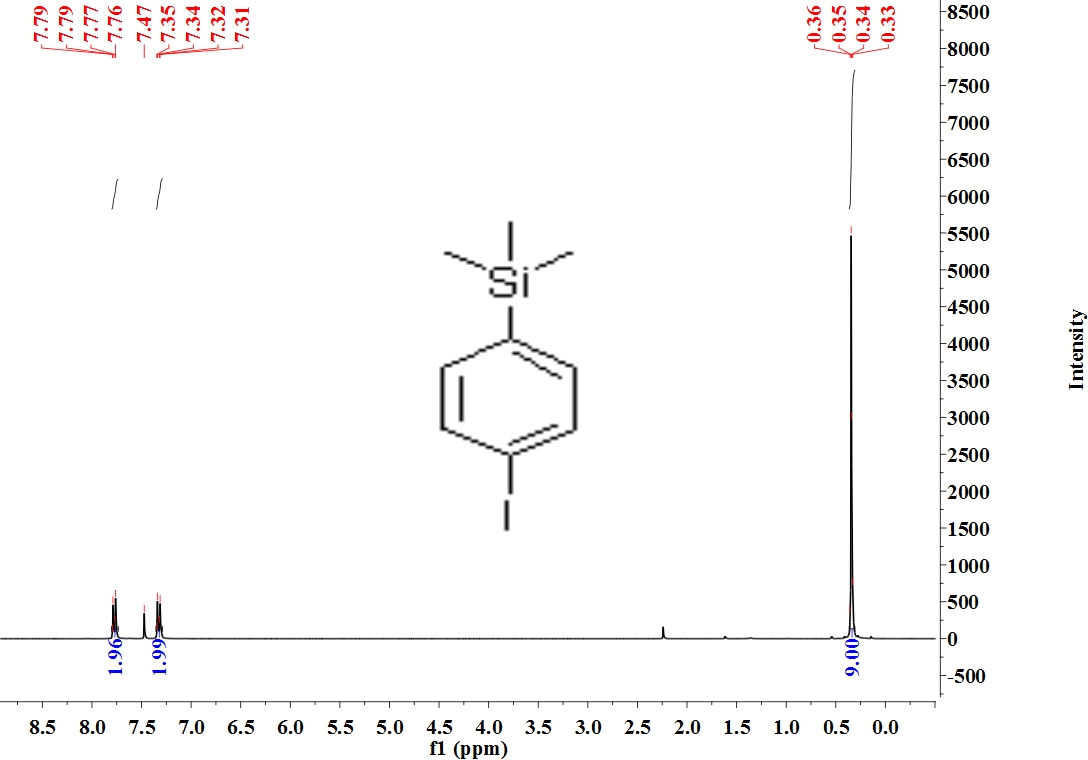


**Supplementary Figure 72.** ^1^H NMR of substrate **17**.


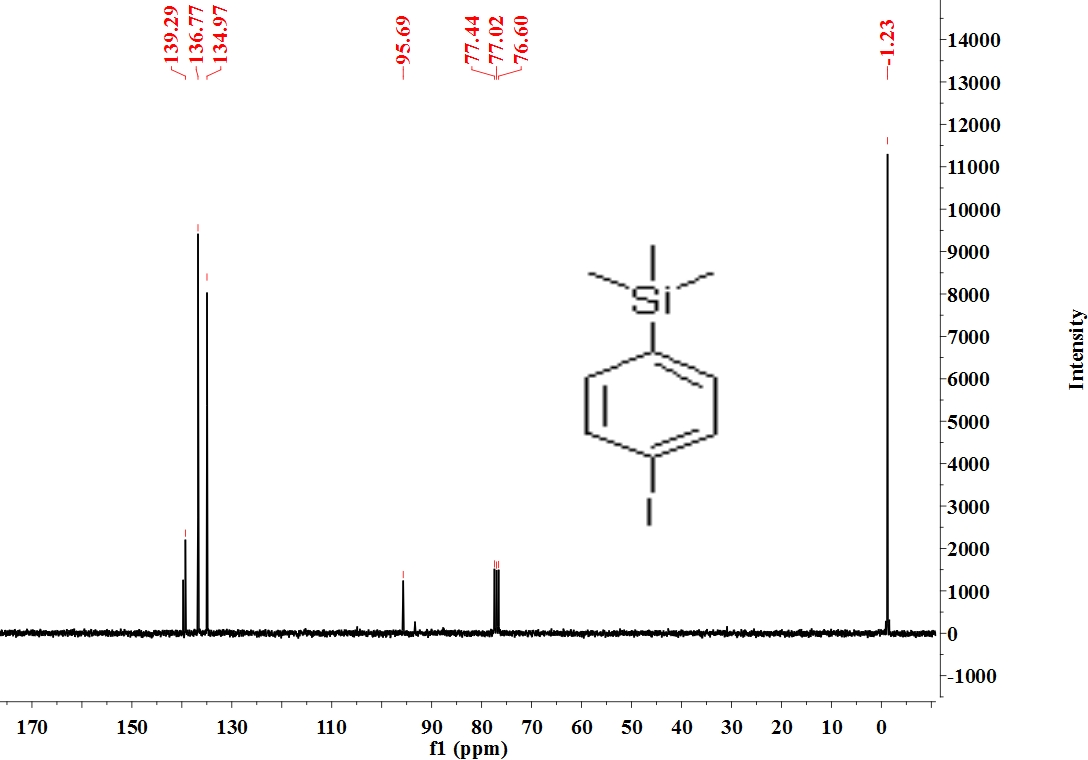


**Supplementary Figure 73.** ^13^C NMR of substrate **17**.

**Supplementary Figure 74.** Synthesis of substrate **18**.


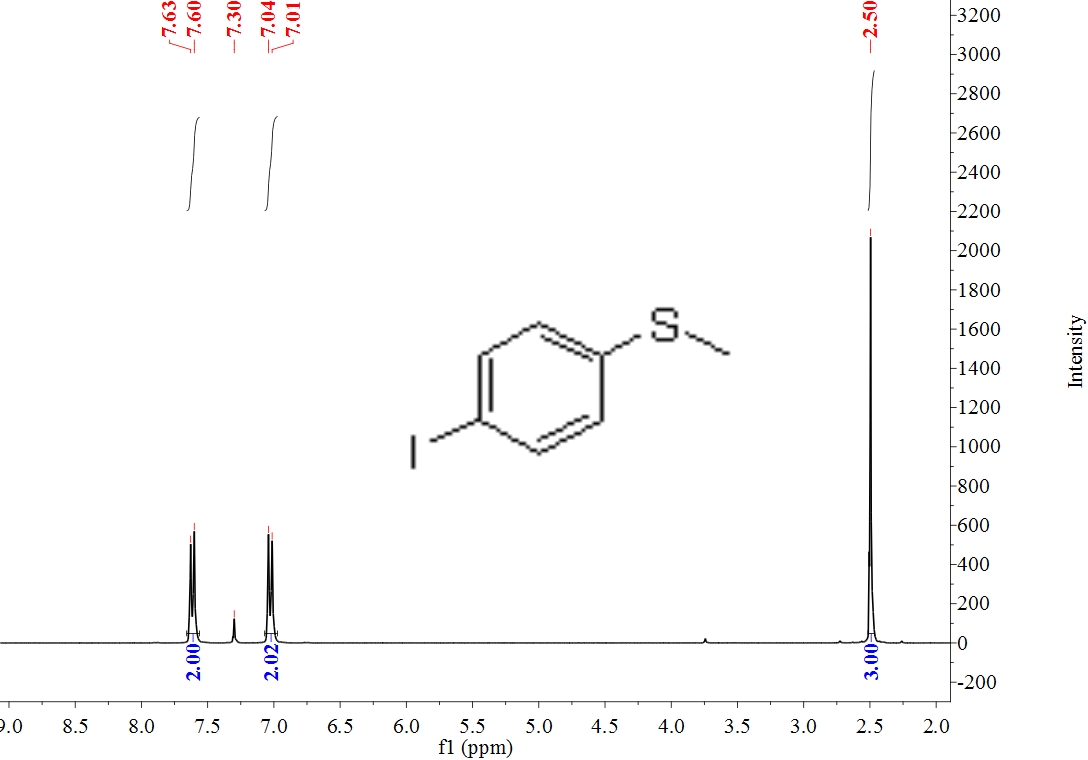


**Supplementary Figure 75.** ^1^H NMR of substrate **18**.


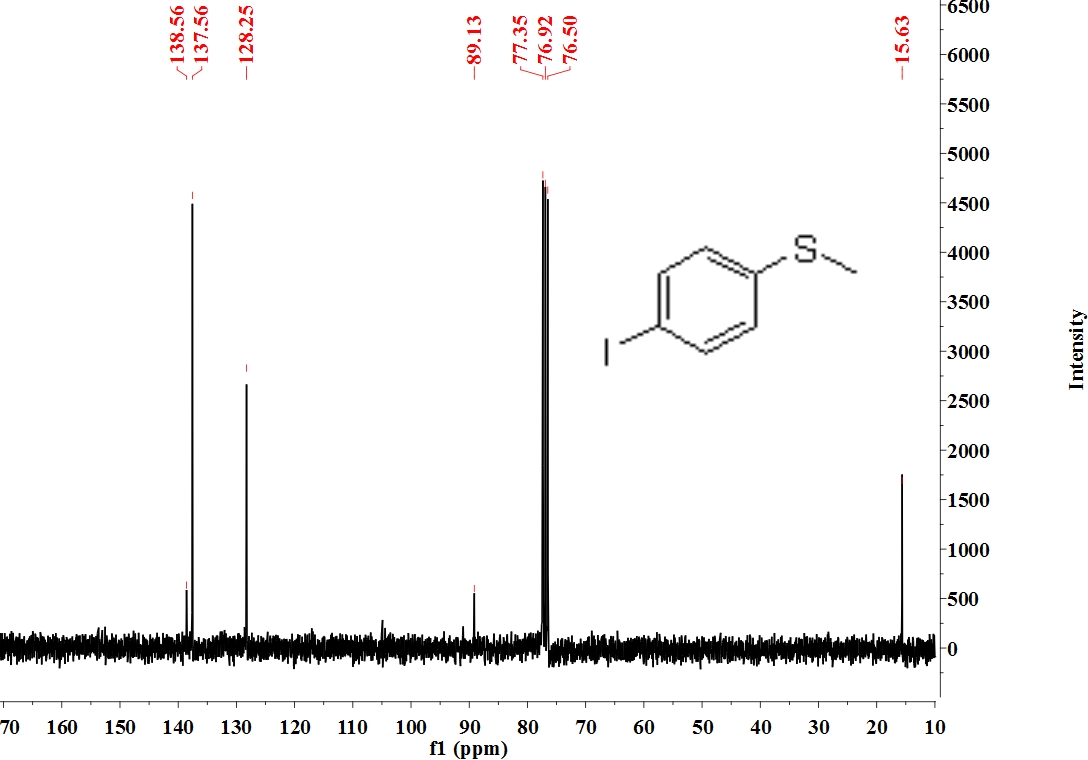


**Supplementary Figure 76.** ^13^C NMR of substrate **18**.

**Supplementary Table 1.** Comparison between H/D exchange and C-X mediated deuteration.

| Approach | **H/D exchange**^4-7^ | | **C-X mediated deuteration** | |
| --- | --- | --- | --- | --- |
|  | Acid/base catalysis | Metal catalysis | Traditional^8^ | Our approach |
| Reaction | C-H to C-D | C-H to C-D | (C-H to C-X) and C-X to C-D | (C-H to C-X) and C-X to C-D |
| C-D Site | Only H with acidity or basicity | Depends on catalyst | Depends on halogen installation and catalyst | Depends on halogen installation |
| Selectivity | Usually full deuteration | Depends on catalyst | Depends on C-X; Most likely C-Br or C-Cl | C-I selective; precise location and number of deuterium |
| Efficiency | Required multiple cycles | Medium to very high | High | High (up to 98% D) |
| Temperature | r.t. to reflux | Elevated temperature | 0 ^o^C or even -78 ^o^C | r.t. |
| Catalyst | Strong acids or bases | Noble metals with/without ligands | Grignard reagents or noble metals | Non-noble photocatalyst without ligands |
| Reagent | D_2_SO_4_ or NaOD (representative) | D_2_ or D_2_O (representative) | ROD, DCOONa or D_2_O (rare) | D_2_O |
| Functional group/Scope | Limited due to strong acids or bases | Medium to wide due to high temperature and noble metals | Medium due to harsh reaction condition | Wide, depends on the installation of halogens on target molecules ^a)^ |
| Advantage | Low-cost, scalability | Synthesis of chiral molecule^9^ | Able for special C_sp2_-X deuteration | Selectivity and wide substrate scope |
| Limitation | Effective to limited substrates. Poor selectivity | Noble metal at high loadings; Toxicity of homogeneous catalysts | Pre-halogenation, harsh condition and special deuteration reagents | Pre-halogenation of C-H bond; Not applicable to chiral molecule |

^a)^ –OH, -NH_2_, -CHO, -COCH_3_, COOCH_3_, -CN, -C=C and -C≡C groups can be retained, which are challenging in C-H/C-D exchange.

**Supplementary Table 2.** Bond dissociation energy for C-H and C-X bonds^10,11^.

| C-H Bond | H_3_C-H | C_2_H_5_-H | (CH_3_)_3_C-H | CH_2_CH-H | HC_2_-H | C_6_H_5_-H | CH_2_CHCH_2_-H |
| --- | --- | --- | --- | --- | --- | --- | --- |
| Bond Energy (kcal mol^-1^) | 105 | 101 | 96.5 | 111 | 133 | 113 | 89 |
| C-X Bond | Ar-I | Ar-Br | Ar-Cl | Ar-F | H_3_C-I | H_3_C-Br | H_3_C-Cl |
| Bond Energy (kcal mol^-1^) | 67 | 84 | 97 | 127 | 56 | 70 | 84 |

**Supplementary Table 3.** Statistics of porous CdSe nanosheets.

|  | Crystalline | Amorphous | Nanopores |
| --- | --- | --- | --- |
| Domain Size (nm) | 1.6 ~ 4.5 | N. A. | 1.2 ~ 2.5 |
| Percentage (area%) | 57 ~ 65% | 14 ~ 18% | 19 ~ 27% |

**Supplementary Table 4.** GC-MS yields for the photocatalytic hydrodehalogenation with or without CdSe photocatalysts under UV light (> 280 nm). UV degradation has negligible influence on the photocatalytic reaction.

| UV light, 2 h |  |  |  |  |
| --- | --- | --- | --- | --- |
| Without CdSe | < 3% | < 3% | < 3% | No reaction |
| With CdSe | > 99% | > 99% | 96% | 63% |
| UV light, 2 h |  |  |  |  |
| Without CdSe | < 3% | No reaction | No reaction | No reaction |
| With CdSe | > 99% | 90% | > 99% | > 99% |

**Supplementary Table 5.** Control experiments on photocatalytic hydrodehalogenation reaction.^a)^

| Entry | Catalyst | Loading | Additive | Solvent | Time | Conversion | GC Yield |
| --- | --- | --- | --- | --- | --- | --- | --- |
| 1 | Porous 2D CdSe | 5 mg | Na_2_SO_3_ | CH_3_CN/H_2_O | 2 h | > 99% | > 99% |
| 2 | Porous 2D CdSe | 5 mg | Na_2_SO_3_ | CH_3_CN/H_2_O | 1 h | 64% | 64% |
| 3 | CdSe nanoparticles | 5 mg | Na_2_SO_3_ | CH_3_CN/H_2_O | 2 h | 36% | 36% |
| 4 ^b)^ | CdSe nanoplates | 5 mg | Na_2_SO_3_ | CH_3_CN/H_2_O | 2 h | trace | trace |
| 5 ^c)^ | CdS powders | 5 mg | Na_2_SO_3_ | CH_3_CN/H_2_O | 2 h | 28% | 28% |
| 6 ^d)^ | TiO_2_ nanopowders | 5 mg | Na_2_SO_3_ | CH_3_CN/H_2_O | 2 h | trace | trace |
| 7 ^e)^ | Porous 2D CdSe | 5 mg | Na_2_SO_3_ | CH_3_CN/H_2_O | 2 h | trace | trace |
| 8 | -- | -- | Na_2_SO_3_ | CH_3_CN/H_2_O | 2 h | trace | trace |
| 9 | Porous 2D CdSe | 5 mg | -- | CH_3_CN/H_2_O | 2 h | 27% | 27% |
| 10 ^f)^ | Porous 2D CdSe | 5 mg | OA, OLA | CH_3_CN/H_2_O | 2 h | 40% | 40% |
| 11 ^g)^ | Porous 2D CdSe | 5 mg | Na_2_SO_3_ | CH_3_CN/H_2_O | 2 h | trace | trace |
| 12 ^h)^ | Porous 2D CdSe | 5 mg | Na_2_SO_3_ | CH_3_CN/H_2_O | 8 h | > 99% | > 99% |

^a)^ Standard conditions: **1c** (0.1 mmol), CdSe nanosheets (5.0 mg), Na_2_SO_3_ (0.25 M), CH_3_CN/H_2_O (2.5 mL/2.5 mL), irradiation 2 h (> 280 nm), r.t.; ^b)^ CdSe nanoplates according to the reported method^12^; ^c)^ Commercial CdS powders from Sigma Aldrich (208183); ^d)^ Commercial TiO_2_ nanopowders from Sigma Aldrich (718467); ^e)^ In the dark; ^f)^ 10.0 mmol OA and 10.0 mmol OLA instead of Na_2_SO_3_, OA = octylamine, OLA = oleylamine; ^g)^ In the dark under 1 balloon of H_2_ for 24 h; ^h)^ > 420 nm.

**Supplementary Table 6.** A comparison in the sacrificing agents for hydrodehalogenation and deuteration.

| Sacrificing Agent | Na_2_SO_3_ | EtN_3_ | MeOH | Na_2_S·9H_2_O | Lactic acid | Na_2_SO_3_ | Na_2_SO_3_ |
| --- | --- | --- | --- | --- | --- | --- | --- |
| Hydrogenation Yield ^a)^ | > 99 % | > 99 % | 0 % | 27 % | 27 % | 94 % ^c)^ | 88 % ^d)^ |
| Deuteration Yield ^b)^ | > 99 % | > 99 % | 0 % | 28 % | 0 % | -- | -- |
| Deuteration Efficiency ^b)^ | 81 % | 49 % | 0 % | 74 % | 0 % | -- | -- |

^a)^ Reaction condition: CH_3_CN/H_2_O, 0.25 M sacrificing agents, *hv* > 280 nm, r.t., 2 h; ^b)^ CH_3_CN/D_2_O, 0.25 M sacrificing agents, *hv* > 420 nm, r.t., 8 h; ^c)^ 0.125 M; ^d)^ 0.0625 M.

**Supplementary Note 1**

The chemical composition of porous CdSe nanosheets was examined from X-ray photoelectron spectroscopy (XPS) in Supplementary Figure 5a. The Cd_3d_ spectrum is fitted with four Gaussian peaks at 405.0, 406.2, 411.7 and 412.9 eV, corresponding to the 3d_5/2_ of CdSe, 3d_5/2_ of CdO, 3d_3/2_ of CdSe and 3d_3/2_ of CdO species, respectively^13,14^. The atomic percentage of CdO species is around 18 atom% due to the partial oxidation of active Cd atoms on porous nanosheets. The peaks located at 53.9 and 54.7 eV in Se_3d_ spectrum are associated to the 3d_5/2_ and 3d_3/2_ of CdSe species.

**Supplementary Note 2**

The powder XRD pattern in Supplementary Figure 5b shows the characteristic diffraction peaks of hexagonal wurtzite-type CdSe and agrees well with the standard pattern (JCPDS No. 08-0459)^13,14^. The peak of {002} plane is particularly strong in comparison with other planes, which indicates the CdSe nanosheets have a preferred {002} orientation due to its layered structure.

**Supplementary Note 3**

We performed thiol functionalization to cap the active Cd sites on CdSe^15^. Such functionalization, or so-called surface passivation, can effectively reduce its activity if active Cd sites are responsible for catalytic reactions. We chose a highly reactive thiol molecule, 3-mercaptopropionic acid (MPA) to fully bind with Cd atoms on porous CdSe nanosheets. The successful functionalization of CdSe is indicated by the characteristic peaks at 3,443, 2,920, 2,851 and 1,634 cm^-1^ in the FT-IR spectra (Supplementary Figure 10b), corresponding to the absorption bands of carboxylic acid groups, asymmetric and symmetric stretching modes of CH_2_ of the MPA molecules and the vibration of carbonyl groups, respectively^16^. Thiol-CdSe only shows much worse catalytic activity towards photocatalytic hydrodehalogenation (63% versus 99%), suggesting a large portion of active sites have been deactivated after thiol functionalization.

In addition to direct functionalization of porous CdSe nanosheets, we also employed another surface passivation technique by using excess amount of capping agents in solution to deactivate those active sites. As shown in Supplementary Figure 13b, Amine-CdSe only possesses inferior activity (68%) after the addition of two small amine ligands (20 mmol amines versus 0.026 mmol CdSe). The physical absorption of oleylamine and octylamine is confirmed by the FT-IR characteristic peaks at 3,258, 3,213, 2,920 and 2,851 cm^-1^ for the N-H vibration of primary amines and the CH_2_ stretching of the long chain backbone. A NH_2_ bending peak at 1,583 cm^-1^ and a C-N stretching vibration at 1,466 cm^-1^ can be also observed in Amine-CdSe. In this regard, both surface passivation techniques can lead to the deactivation of porous CdSe nanosheets, which suggests the active Cd sites are responsible for photocatalytic reactions.

**Supplementary Methods**

**Synthesis of porous, 2D CdSe**: CdSe nanosheets were synthesized according to the reported method with some modifications to introduce nanoporous structure^17^. Briefly, 1.5 mmol (0.275 g) of anhydrous CdCl_2_ was dissolved in 10 mL of oleylamine and octylamine mixture (1:1 v/v) at 120 ^o^C for 2 hours under argon and then cooled down to r.t. 4.5 mmol (0.355 g, Se : Cd = 3 : 1) of selenide was dispersed in 5 mL of oleylamine and octylamine mixture (1:1 v/v) at r. t. with vigorous stirring. The selenide dispersion was then injected into Cd-containing solution at r.t. The resulting mixture was heated to 100 °C for 16 hours under argon, with an obvious color change from black to green (too early), turbid yellow (end point) and red (overshoot). The nanosheets were precipitated by adding excess amount of ethanol and the powders were redispersed in CHCl_3_ for further purification. We didn’t add trioctylphosphine to remove the excess amount of selenide powders. Most of selenide could be removed after standing for 5 mins prior to any treatment. Low speed centrifugation was also used for the removal of remaining selenide powders. The crude CdSe nanosheets were then purified with a silica column (Mobile phase: CHCl_3_. Stationary phase: reagent-grade silica with a pore size of 90 Å, a particle size of 100-200 mesh, a surface ratio of 300-400 m^2^g^-1^ and a pH value of 6.5-7.5, from Sanpont). This was found to be a key step for the preparation of porous CdSe nanosheets. Porous CdSe solids were then recovered by rotary evaporation of the CHCl_3_ dispersion at r.t. under vacuum.

**General procedure for photocatalytic H_2_ evolution**: Experiments were performed using 10.0 mg of photocatalysts in 30 mL of 0.5 M Na_2_SO_3_ /Na_2_S aqueous solution under UV-light irradiation (λ > 280 nm). The amount of H_2_ evolution was monitored by PGS-15 auto-sampling device and Agilent 7890A-GC. The H_2_ peak was calibrated by the injection of pure H_2_ gas to obtain the working curve.

**General procedure for photocatalytic hydrodehalogenation of halides**: The standard procedure is illustrated here using 4-iodoanisole (**1c**) as an example. All conditions were kept constant unless further specifications were provided. Typically, 5 mg of porous CdSe nanosheets, 0.1 mmol of 4-iodoanisole (**1c**) and Na_2_SO_3_ (0.25 M) were dispersed in a CH_3_CN/H_2_O mixture (2.5 mL/2.5 mL) and sonicated for 30 min. The reaction mixture was then irradiated with a Xenon lamp (150 W, λ > 280 nm) for 2 h or visible light (> 420 nm with a cut-off filter) for 6 to 8 h at r.t. under argon. After reaction, the mixture was centrifuged to remove photocatalyst. The supernatant was extracted by adding 5 mL of CH_2_Cl_2_ and the organic phase was analysed by GC-MS. The yields were calculated from standard calibration curves. Isolated product was obtained by silica column chromatography. The yield was calculated by dividing the amount of the obtained desired product by the theoretical yield. 2D-CdSe catalyst can be recovered by simple filtration and rising with CH_3_CN.

**Optical measurements:** Time-resolved transient absorption and pump-probe measurements were performed on porous CdSe dispersion in CHCl_3_ by using a Ti:sapphire oscillator seeded regenerative amplifier (Spectra Physics Spitfire pro), which gives an output with a pulse energy of 2.0 mJ at 800 nm and a repetition rate of 1 kHz. The 800 nm laser beam was split into two portions. The larger portion of the beam passed through a BBO crystal to generate the 400 nm pump beam by second-harmonic generation. A small portion of the 800 nm beam was focused onto a 1 mm sapphire plate to generate a white light continuum. The pump beam was focused onto the sample with a beam size of 300 μm and overlapped with the smaller diameter (200 μm) probe beam. The delay between the pump and probe pulses was varied by a computer-controlled translation stage (Newport, ESP 300). The pump beam was modulated by an optical chopper at a frequency of 500 Hz. The variation transmittance at selected probe wavelength was recorded as a function of time delay between pump and probe pulses. All of the experiments were performed at r.t. in a 1 mm path length cuvette. During the measurements, the extinction spectra of the sample were checked before and after the experiments, and only a subtle difference was observed, which suggests that the sample is stable and there is little photo-damage to the sample during the course of the experiments.

**Electrochemical measurements:** Cyclic voltammograms (CVs) were recorded on an Autolab PGSTAT30 digital potentiostat /galvanostat at r.t., using a glassy carbon electrode (3 mm in diameter), a Pt wire and an Ag/Ag^+^ electrode (Ag wire with 10 mM AgNO_3_ in CH_3_CN) as the working, counter and reference electrodes, respectively. The glassy carbon electrode was polished with 3 μm, 1 μm diamond and 0.05 μm alumina powders before use. The scan rate was set as 20 mV s^-1^ and the electrolyte solution was thoroughly deoxygenated by bubbling high purity argon for 15 mins prior to and during all the experiments.

**Bromobenzene-4-*d* for Suzuki coupling reaction**^18^**:** After the photocatalytic deuteration of 1-bromo-4-iodobenzene, the reaction mixture was centrifuged and the solution containing the deuterated product bromobenzene-4-*d* was used directly for the subsequent Suzuki coupling reaction without purification. Phenylboronic acid (99%, 14.8 mg, 0.12 mmol, 1.2 equiv), Pd(dppf)Cl_2_·DCM (99%, 8.2 mg, 0.01 mmol, 0.1 equiv) and K_3_PO_4_ (63.7 mg, 0.3 mmol, 3 equiv) was added to a 25 mL Schlenk tube. Then, a solution of THF/D_2_O (2.5 mL/1.5 mL) containing 1-bromobenzene-4-*d* (**2ak**) was added. The reaction mixture was then heated to 70 °C under argon for 18 h before cooling down to r.t. The reaction mixture was then diluted with EtOAc (30 mL) and washed with brine (30 mL). The organic phase was dried by anhydrous sodium sulfate and then removed under reduced pressure. After purification by chromatography on silica, product **3** was obtained with **M** = 9.8 mg. Yield = 63%, white solid, Deuterated ratio = 81%, **^1^H NMR** (300 MHz, Chloroform-*d*) *δ* [ppm] 7.67 - 7.61 (m, 4H), 7.49 (tt, *J* = 6.2, 1.3 Hz, 4H), 7.43 - 7.38 (m, 1H), 7.37 (t, *J* = 1.3 Hz, 0.19H); **^13^C NMR** (300 MHz, Chloroform-*d*) *δ* [ppm] 141.2, 128.6, 128.5, 127.1, 127.0; **GCMS** (EI) 155.1, theoretical value for C_12_H_9_D 155.1.

**(Phenyl-4-d)boronic acid for Suzuki coupling reaction**^18^**:** After the photocatalytic deuteration of (4-iodophenyl)boronic acid, the reaction mixture was centrifuged and the solution containing the deuterated product (phenyl-4-*d*)boronic acid was used directly for next Suzuki coupling reaction without purification. Bromobenzene (99%, 19 mg, 0.12 mmol, 1.2 equiv), Pd(dppf)Cl_2_·DCM (99%, 8.2 mg, 0.01 mmol, 0.1 equiv) and K_3_PO_4_ (63.7 mg, 0.3 mmol, 3 equiv) was added a 25 mL Schlenk tube. Then, a solution of DMF/D_2_O (2.5 mL/1.5 mL) containing (phenyl-4-d)boronic acid (**2al**) was injected to the system. The reaction mixture was then heated to 70 °C under argon for 18 h before cooling down to r.t. The reaction mixture was then diluted with EtOAc (30 mL) and washed with brine (30 mL). The organic phase was dried by anhydrous sodium sulfate and then removed under reduced pressure. After purification by chromatography on silica, product **4** was obtained with **M** = 10.4 mg. Yield = 67%, white solid, Deuterated ratio = 80%, **^1^H NMR** (300 MHz, Chloroform-*d*) *δ* [ppm] 7.68 - 7.62 (m, 4H), 7.54 - 7.46 (m, 4H), 7.44 - 7.39 (m, 1H), 7.37 (t, *J* = 1.3 Hz, 0.20H); **^13^C NMR** (300 MHz, Chloroform-*d*) *δ* [ppm] 141.2, 128.6, 128.5, 127.1, 127.0; **GCMS** (EI) 155.1, theoretical value for C_12_H_9_D 155.1.

**Copper-catalyzed Click reaction of deuterium-containing alkyl with (azidomethyl) benzene**^19^**:** 1-(but-3-yn-1-yl)benzene-4-*d* (13.1 mg, 0.10 mmol, **2am**) and (azidomethyl) benzene (17.3 mg, 0.13 mmol) were added to a solution of CH_3_CN (5.0 mL) containing Cu(OAc)_2_**·**H_2_O (1.9 mg, 0.01 mmol, 0.1 equiv). The resulting mixture was stirred at r.t. for 12 h. After reaction, the solvent was removed and the residues were purified by a flash chromatography, which furnished the desired product **5**. **M** = 22.6 mg, yield = 85%, white solids, Deuterated ratio = 78%, **^1^H NMR** (300 MHz, Acetonitrile-*d*_3_) *δ* [ppm] 7.87 (s, 1H), 7.45 - 7.42 (m, 0.22H), 7.42 - 7.35 (m, 3H), 7.35 - 7.30 (m, 4H), 7.03 (d, *J* = 2.0 Hz, 1H), 7.01 (d, *J* = 2.0 Hz, 1H), 5.57 (s, 2H), 5.16 (s, 2H); **^13^C NMR** (300 MHz, Acetonitrile-*d*_3_) *δ* [ppm] 158.3, 143.8, 135.8, 129.4, 129.3, 128.8, 128.3, 127.9, 123.6, 114.7, 61.3, 53.4; **GCMS** (EI) 266.2, theoretical value for C_16_H_14_DN_3_O 266.3.

**Terminal deuterated alkyl for C-H bond insertion reaction**^20^**:** Ethyl diazoacetate (15% in toluene, 27.4 mg of toluene solution, 0.12 mmol, 1.2 equiv) was added to a solution of **2ao** (16.1 mg, 0.1 mmol, 1.0 equiv) and CuI (98%, 1.9 mg, 0.01 mmol, 0.1 equiv) in CH_3_CN (5.0 mL). The resulting mixture was stirred at r.t. for 18 h. Then, the volatile were removed and the residue were purified by a flash chromatography, which furnished the desired product **6**. **M** = 19.8 mg, yield = 80%, yellow oil, Deuterated ratio = 70%, **^1^H NMR** (300 MHz, Chloroform-*d*) *δ* [ppm] 9.93 (s, 1H), 7.88 (d, *J* = 8.6 Hz, 2H), 7.07 (d, *J* = 8.6 Hz, 2H), 5.95 - 5.79 (m, 1.3 H), 4.82 (dd, *J* = 6.3, 2.4 Hz, 2H), 4.31 - 4.16 (m, 2H), 1.31 (t, *J* = 7.1 Hz, 3H); **^13^C NMR** (300 MHz, Chloroform-*d*) *δ* [ppm] 212.2, 190.5, 164.8, 162.9, 132.2, 131.8, 130.4, 115.1, 91.9, 90.5, 64.3, 61.1, 14.1; **GCMS** (EI) 247.1, theoretical value for C_14_H_13_DO_4_ 247.3.

**Synthesis of substrate 7:** Substrate **7** was synthesized according to the reported procedure^21^. To a solution of PPh_3_ (1.1 g, 4.2 mmol) and imidazole (0.29 g, 4.2 mmol) in CH_2_Cl_2_ (10 mL) was carefully added iodine (2.1 g, 4.2 mmol) at 0 °C (exothermic reaction). After 15 min, a solution of alcohol (1.1 g, 4.0 mmol) in CH_2_Cl_2_ (4 mL) was added dropwise (exothermic reaction). The ice bath was removed and the suspension was stirred overnight. The CH_2_Cl_2_ was almost completely removed under reduced pressure to provide an orange slurry which was then diluted with 40 mL pentane and filtered through a pad of celite. The solvents were removed under reduced pressure and purified by chromatography on silica to obtain substrate **7**. **M** = 1.2 g, yield = 78%, yellow oil, **^1^H NMR** (300 MHz, Chloroform-*d*) *δ* [ppm] 7.58 (s, 2H), 7.00 (s, 2H), 4.05 (s, 2H), 3.27 (s, 2H), 1.88 (dt, *J* = 13.0, 6.5 Hz, 4H), 1.53 (s, 4H); **^13^C NMR** (300 MHz, Chloroform-*d*) *δ* [ppm] 161.4, 126.8, 126.6, 126.6 (q, *J* = 430 Hz), 114.3, 67.9, 33.2, 30.1, 28.8, 24.9, 6.7; **GCMS** (EI) 372.0, theoretical value for C_13_H_16_F_3_IO 372.2.

**Synthesis of substrate 8:** To a solution of *N*-(4-iodophenyl)acetamide (1.1 g, 4.0 mmol) in 15 mL of DMF, 1.0 g of 1-iodohexane (4.8 mmol) was added, followed by the addition of 629.4 mg of potassium *tert*-butoxide (4.4 mmol) in portions. The reaction mixture was stirred overnight at 70 °C. After cooling to r.t., water (20 mL) was added and then the mixture was extracted by CH_2_Cl_2_ (50 mL × 3). The combined organic layers were dried over anhydrous MgSO_4_ and evaporated *in vacuo*. The residues were purified by chromatography on silica to obtain substrate **8**. **M** = 1.3 g, yield = 90%, light purple oil, **^1^H NMR** (300 MHz, Chloroform-*d*) *δ* [ppm] 7.78 (d, *J* = 8.5 Hz, 2H), 6.94 (d, *J* = 8.5 Hz, 2H), 3.67 (d, *J* = 15.2 Hz, 2H), 1.86 (s, 3H), 1.56 - 1.41 (m, 2H), 1.27 (s, 6H), 0.88 (t, *J* = 6.5 Hz, 3H); **^13^C NMR** (300 MHz, Chloroform-*d*) *δ* [ppm] 169.8, 142.8, 138.8, 130.0, 92.9, 49.0, 31.4, 27.6, 26.3, 22.7, 22.4, 13.9; **LCMS** (APCI) 346.1, theoretical value for C_14_H_20_INO 346.0.

**Synthesis of substrate 9:** Potassium carbonate (718.6 mg, 5.2 mmol) and 3-bromoprop-1-ene (629.2 mg, 5.2 mmol) was slowly added to a solution of 2-iodophenol (880.0 mg, 4.0 mmol) in 15 mL of DMF at r.t. The reaction mixture was stirred overnight at 70 °C, quenched by careful addition of water (20 mL) and extracted with CH_2_Cl_2_ (50 mL × 3) after cooling down to r.t. The combined organic layers were dried over anhydrous MgSO_4_ and evaporated *in vacuo*. The residues were purified by chromatography on silica to obtain substrate **9**. **M** = 894.7 mg, yield = 86%, light yellow oil, **^1^H NMR** (300 MHz, Chloroform-*d*) *δ* [ppm] 7.92 - 7.77 (m, 1H), 7.43 - 7.25 (m, 1H), 6.85 (d, *J* = 8.2 Hz, 1H), 6.83 - 6.71 (m, 1H), 6.24 - 6.01 (m, 1H), 5.57 (dq, *J* = 17.2, 1.6 Hz, 1H), 5.46 - 5.31 (m, 1H), 4.64 (dt, *J* = 4.6, 1.6 Hz, 2H); **^13^C NMR** (300 MHz, Chloroform-*d*) *δ* [ppm] 157.0, 139.4, 132.5, 129.3, 122.6, 117.5, 112.5, 86.6, 69.6; **HRMS** (APCI) 259.9683, theoretical value for C_9_H_9_IO 259.9698.

**Synthesis of substrate 11:** Substrate **11** was synthesized according to the reported procedure^22^. Fine powders of CuO (0.8 g, 10.0 mmol) and I_2_ (2.53 g, 10.0 mmol) were added into a solution of aryl methyl ketone (1.2 g, 10.0 mmol) in anhydrous MeOH (20 mL). The mixture was refluxed for 2 h. After the disappearance of aryl methyl ketone (monitored by TLC), the mixture was filtered and the solvent was removed under reduced pressure. The residue was poured into 10% Na_2_S_2_O_3_ solution (50 mL), the mixture was extracted with EtOAc (50 mL × 3), and the organic layer was dried over anhydrous Na_2_SO_4_. The desired substrate **11** was obtained after the removal of the solvent and purification of the residue by column chromatography. **M** = 2.6 g, yield = 95%, purple oil, **^1^H NMR** (300 MHz, Chloroform-*d*) *δ* [ppm] 8.01 (d, *J* = 8.6 Hz, 2H), 6.99 (d, *J* = 8.7 Hz, 2H), 4.35 (s, 2H), 3.92 (s, 3H); **^13^C NMR** (300 MHz, Chloroform-*d*) *δ* [ppm] 191.4, 163.9, 131.3, 126.4, 114.0, 55.5, 1.5; **HRMS** (APCI) 276.9717, theoretical value for C_9_H_9_IO_2_ 276.9647.

**Synthesis of substrate 13:** NaH (221.5 mg, 6.0 mmol) and propargyl bromide (892.5 mg, 7.5 mmol) was added to a solution of 4-hydroxybenzaldehyde (610.5 mg, 5.0 mmol) in DMF (20 mL). The reaction mixture was stirred at r.t. overnight, filtered, and partitioned between EtOAc and H_2_O. The organic phase was washed several times with water, washed once with brine, dried over anhydrous Na_2_SO_4_ and concentrated. The crude product was purified via column chromatography to give pure substrate **13**. **M** = 624.8 mg, yield = 78%, white solid, **^1^H NMR** (300 MHz, Chloroform-*d*) *δ* [ppm] 9.94 (s, 1H), 7.89 (dt, *J* = 9.4, 2.6 Hz, 2H), 7.16 - 7.10 (dt, *J* = 9.4, 2.6 Hz, 2H), 4.82 (d, *J* = 2.4 Hz, 2H), 2.61 (t, *J* = 2.4 Hz, 1H); **^13^C NMR** (300 MHz, Chloroform-*d*) *δ* [ppm] 190.7, 162.3, 131.8, 130.5, 115.1, 77.4 (d, *J* = 12.0 Hz), 76.2 (d, *J* = 15.0 Hz), 55.9; **GCMS** (EI) 160.0, theoretical value for C_10_H_8_O_2_ 160.2.

**Synthesis of substrate 14:** Substrate **14** was synthesized according to the reported procedure^23^. AgNO_3_ (25.5 mg, 0.15 mmol) was added to the mixture of terminal alkyne (480.6 mg, 3.0 mmol), NIS (810 mg, 3.6 mmol) and 30 mL acetone and the mixture was stirred at r.t. for 3 h. The precipitate was filtered off after reaction. The solvent was evaporated and the residue was purified via column chromatography to give pure substrate **14_._** **M** = 781.1 mg, yield = 91%, light yellow solid, **^1^H NMR** (300 MHz, Chloroform-*d*) *δ* [ppm] 9.94 (d, *J* = 6.0 Hz, 1H), 7.93 - 7.85 (m, 2H), 7.14 - 7.07 (m, 2H), 4.95 (d, *J* = 3.8 Hz, 2H); **^13^C NMR** (300 MHz, Chloroform-*d*) *δ* [ppm] 190.6, 162.3, 131.8, 130.6, 115.1, 88.0, 57.4, 5.9; **HRMS** (APCI) 286.9563, theoretical value for C_10_H_7_IO_2_ 286.9524.

**Synthesis of substrate 15:** Substrate **15** was synthesized according to the reported procedure^24^. 20 mL of CH_2_Cl_2_ containing 1-(3-dimethylaminopropyl) -3-ethylcarbodiimide hydrochloride (EDCI, 920.1 mg, 4.8 mmol) was slowly added to a solution of quinolin-8-amine (576.8 mg, 4.0 mmol), 5-fluoro-2-iodobenzoic acid (1.2 g, 4.4 mmol) and *N*,*N*-dimethyl-4-aminopyridine (DMAP, 0.4 mmol, 48.9 mg) in 15 mL of anhydrous CH_2_Cl_2_ under argon. After stirring at r.t. for 10 h, 30 mL of water was added to quench the reaction. The reaction mixture was extracted with CH_2_Cl_2_ (20 mL × 3) and the combined organic solvent was dried over anhydrous Na_2_SO_4_, filtered and concentrated under reduced pressure. The resulting residue was purified by column chromatography to give pure substrate **15**. **M** = 721.6 mg, yield = 46%, while solid, **^1^H NMR** (300 MHz, Chloroform-*d*) *δ* [ppm] 10.20 (s, 1H), 8.95 (dd, *J* = 6.5, 2.6 Hz, 1H), 8.88 - 8.77 (m, 1H), 8.24 (d, *J* = 8.3 Hz, 1H), 7.94 (dd, *J* = 8.5, 5.4 Hz, 1H), 7.64 (d, *J* = 6.1 Hz, 2H), 7.51 (dd, *J* = 8.1, 4.1 Hz, 1H), 7.46 - 7.35 (m, 1H), 6.99 (td, *J* = 8.3, 2.3 Hz, 1H); **^13^C NMR** (300 MHz, Chloroform-*d*) *δ* [ppm] 166.0 (d, *J* = 6.0 Hz), 164.3, 161.0, 148.3, 143.8 (d, *J* = 27.0 Hz), 141.7 (d, *J* = 27.0 Hz), 138.4, 136.4, 133.9, 127.9, 127.3, 122.4, 121.7, 118.9 (d, *J* = 87 Hz), 117.0, 116.1 (d, *J* = 93 Hz); **LCMS** (APCI) 393.0, theoretical value for C_16_H_10_FIN_2_O 393.0.

**Synthesis of substrate 16:** Substrate **16** was synthesized according to the reported procedure^25^. 20 mL of Et_2_O containing 1,4-diiodobenzene (1.6 g, 5 mmol) was added to 1.8 mL of *n*-butyllithium (3 M in THF, 5.5 mmol) at -78 ºC. The mixture was stirred for 1 h at the same temperature with the addition of chlorodimethylsilane (473 mg, 5 mmol). 15 mL of H_2_O was added after gradually warming to r.t. The mixture was extracted with *n*-hexane (10 mL × 3), and the combined organic extract was washed with brine (15 mL) and dried over anhydrous Na_2_SO_4_. After filtration, the filtrate was concentrated under reduced pressure. The residue was purified by flash column chromatography to give the product **16**. **M** = 996.4 mg, yield = 76%, colorless oil, **^1^H NMR** (300 MHz, Chloroform-*d*) *δ* [ppm] 7.80 - 7.70 (m, 2H), 7.35 - 7.27 (m, 2H), 4.44 (hept, *J* = 3.7 Hz, 1H), 0.38 (d, *J* = 3.8 Hz, 6H); **^13^C NMR** (300 MHz, Chloroform-*d*) *δ* [ppm] 136.9, 136.7, 135.6, 96.0, -4.0; **LCMS** (APCI)262.0, theoretical value for C_18_H_11_ISi 262.2.

**Synthesis of substrate 17:** Substrate 1**7** was synthesized according to the reported procedure^26^. 10 mL of Et_2_O containing 1,4-diiodobenzene (1.6 g, 5 mmol) was added to 1.6 mL of butyllithium (3 M in THF, 5 mmol) at -78 °C. The reaction mixture was stirred for 4 h at the same temperature. After the addition of chlorotrimethylsilane (618 mg, 5.7 mmol), the mixture was stirred for another 10 min at -78 °C. Excess amount of H_2_O was added to the mixture after stirring at r.t. for 2 h, and organic phase was separated. The aqueous phase was extracted with Et_2_O (3 times), the combined organic phase was washed with brine and dried over anhydrous MgSO_4_. The solvents were removed under reduced pressure. The resulting residue was purified by column chromatography to give pure substrate **17**. **M** = 1.1 g, yield = 81%, colorless oil, **^1^H NMR** (300 MHz, Chloroform-*d*) *δ* [ppm] 7.78 (dd, *J* = 8.1, 1.7 Hz, 2H), 7.33 (dd, *J* = 8.1, 1.7 Hz, 2H), 0.34 (d, *J* = 1.1 Hz, 9H); **^13^C NMR** (300 MHz, Chloroform-*d*) *δ* [ppm] 139.3, 136.8, 135.0, 95.7, -1.2; **LCMS** (APCI) 276.0, theoretical value for C_9_H_13_ISi 276.2.

**Synthesis of substrate 18:** Substrate **18** was synthesized according to the reported procedure^27^. 20 mL of DMF containing KI (2 mmol), (4-(methylthio) phenyl)boronic acid (3 mmol), CuBr_2_ (45 mg, 10 mol%) and 1,10-phenanthroline (72 mg, 20 mol %) was added to a sealed reaction tube under O_2_. The mixture was stirred at 80 ^o^C for 20 h. After the completion of the reaction, the solvent was evaporated under reduced pressure and the residue was purified by flash column chromatography on silica gel to give the substrate **18**. **M** = 450.2 mg, yield = 90%, white solid, **^1^H NMR** (300 MHz, Chloroform-*d*) *δ* [ppm] 7.61 (d, *J* = 8.2 Hz, 2H), 7.03 (d, *J* = 8.3 Hz, 2H), 2.50 (s, 3H); **^13^C NMR** (300 MHz, Chloroform-*d*) *δ* [ppm] 138.6, 137.6, 128.2, 89.1, 15.6; **GCMS** (EI) 249.9, theoretical value for C_7_H_7_ISi 250.1.

**Supplementary References**

1. Haram, S. K., Quinn, B. M. & Bard, A. J. Electrochemistry of CdS nanoparticles: A correlation between optical and electrochemical band gaps. *J. Am. Chem. Soc.* **123**, 8860-8861 (2001);
2. Zhong, H. *et al.* Noninjection gram-scale synthesis of monodisperse pyramidal CuInS_2_ nanocrystals and their size-dependent properties*.* *ACS Nano* **4**, 5253-5262 (2010);
3. Wang, J. J. *et al.* Photocatalytic hydrogen evolution from glycerol and water over nickel -hybrid cadmium sulfide quantum dots under visible-light irradiation. *ChemSusChem* **7**, 1468-1475 (2014);
4. Atzrodt, J., Derdau, V., Fey, T. & Zimmermann, J. The renaissance of H/D exchange. *Angew. Chem. Int. Ed.* **46**, 7744-7765 (2007);
5. Junk, T. & Catalls, W. J. Hydrogen isotope exchange reactions involving C-H (D, T) bonds. *Chem. Soc. Rev.* **26**, 401-406 (1997);
6. Konermann, L., Pan, J. X. & Liu, Y.-H. Hydrogen exchange mass spectrometry for studying protein structure and dynamics. *Chem. Soc. Rev.* **40**, 1224-1234 (2011);
7. Alonso, F., Beletskaya, I. P. & Yus, M. Metal-mediated reductive hydrodehalogenation of organic halides. *Chem. Rev.* **102**, 4009- 4092 (2002);
8. Munz, D. *et al.* Proton or metal? The H/D exchange of arenes in acidic solvents. *ACS Catal.* **5**, 769-775 (2015);
9. Hale, L. V. A. & Szymczak, N. K. Stereoretentive deuteration of *α*-chiral amines with D_2_O. *J. Am. Chem. Soc.* **138**, 13489-13492 (2016);
10. Blanksby, S. J. & Ellison, G. B. Bond dissociation energies of organic molecules. *Acc. Chem. Res.* **36**, 255-263 (2003);
11. Weaver, J. & Senaweera, S. C-F activation and functionalization of perfluoro- and polyfluoroarenes. *Tetrahedron* **70**, 7413-7428 (2014);
12. Yu, Y., Zhang, J., Wu, X., Zhao, W. & Zhang, B. Nanoporous single-crystal-like Cd_x_Zn_1−x_S nanosheets fabricated by the cation-exchange reaction of inorganic-organic hybrid ZnS- amine with cadmium ions. *Angew. Chem. Int. Ed.* **51**, 897-900 (2012);
13. Chen, C. Y. *et al.* Type-II CdSe/CdTe/ZnTe (core-shell-shell) quantum dots with cascade band edges: The separation of electron (at CdSe) and hole (at ZnTe) by the CdTe layer. *Small* **1**, 1215-1220 (2005);
14. Kim, H. B. & Jang, D. J. Dislocation-driven growth of porous CdSe nanorods from CdSe (ethylenediamine)_0.5_ nanorods. *Nanoscale* **8**, 403-410 (2016);
15. Chou, S. S. *et al.* Ligand conjugation of chemically exfoliated MoS_2_. *J. Am. Chem. Soc.* **135**, 4584-4587 (2013);
16. Chen, Z. & Lu, H. Constructing sacrificial bonds and hidden lengths for ductile graphene /polyurethane elastomers with improved strength and toughness. *J. Mater. Chem.* **22**, 12479-12490 (2012);
17. Son, J. S. *et al.* Large-scale soft colloidal template synthesis of 1.4 nm thick CdSe nanosheets. *Angew. Chem. Int. Ed.* **48**, 6861-6864 (2009);
18. Fyfe, J. W. B., Fazakerley, N. J. & Watson, A. J. B. Chemoselective Suzuki–Miyaura cross-coupling via kinetic transmetallation. *Angew. Chem. Int. Ed.* **56**, 1249-1253 (2017);
19. Cao, L. M., Liu, C. B., Tang, X. Y., Yin, X. G. & Zhang, B. Highly selective synthesis of 1-polyfluoroaryl-1,2,3-triazoles via a one-pot three-component reaction. *Tetrahedron Lett.* **55**, 5033-5037 (2014);
20. Suárez, A. & Fu, G. C. A Straightforward and mild synthesis of functionalized 3-alkynoates. *Angew. Chem. Int. Ed.* **43**, 3580-3582 (2004);
21. Li, D. P., Pan, X. Q., An, L. T., Zou, J. P. & Zhang, W. Manganese(III)-mediated selective diphenylphosphinoyl radical reaction of 1,4-diaryl-1-butynes for the synthesis of 2‑phosphinoylated 3,4-dihydronaphathalenes. *J. Org. Chem.* **79**, 1850-1855 (2014);
22. Zhu, Y. P. *et al.* Target-oriented synthesis: miscellaneous synthetic routes to access 1,4-enediones through the coupling of 1,3-dicarbonyl compounds with multiform substrates. *Tetrahedron* **69**, 6392-6398 (2013);
23. Chen, Z. W., Jiang, H. F., Li, Y. B. & Qi, C. R. Highly efficient two-step synthesis of (*Z*)-2-halo-1-iodoalkenes from terminal alkynes. *Chem. Commun.* **46**, 8049-8051 (2010);
24. Zhang, L. B. *et al.* Cobalt (II)-catalyzed C–H amination of arenes with simple alkylamines. *Org. Lett.* **18**, 1318-1321 (2016);
25. Itami, K., Terakawa, K., Yoshida, J.-I. & Kajimoto, O. Efficient and rapid C-Si bond cleavage in supercritical water. *J. Am. Chem. Soc.* **125**, 6058-6059 (2003);
26. Yoshida, S., Shimomori, K., Kim, Y. & Hosoya, T. Single C-F bond cleavage of trifluoromethylarenes with an ortho-silyl group. *Angew. Chem. Int. Ed.* **55**, 10406-10409 (2016);
27. Zhang, G. Y., Lv, G. L., Li, L. P., Chen, F. & Cheng, J. Copper-catalyzed halogenation of arylboronic acids. *Tetrahedron Lett.* **52**, 1993-1995 (2011).
